# Articulated Untethered Magnetic Actuators for Multimodal and Cross-Scale Operations

**DOI:** 10.34133/cbsystems.0560

**Published:** 2026-05-19

**Authors:** Zhixian Chen, Xiaoyu Zhao, Ying Liu, Shengli Mi

**Affiliations:** ^1^ Bio-manufacturing Engineering Laboratory, Institute of Data and Information, Tsinghua Shenzhen International Graduate School, Tsinghua University, Shenzhen 518000, China.; ^2^ Center of Double Helix, Tsinghua Shenzhen International Graduate School, Tsinghua University, Shenzhen 518000, China.

## Abstract

Untethered magnetic actuators hold great promise for minimally invasive medicine, yet reconciling functional versatility with simple, reliable control remains a critical challenge. To address this, we introduce a modular design paradigm that employs revolute joints to interconnect discrete rigid modules. This strategy synergizes the mechanical robustness of rigid systems with the reconfigurability of soft robots, imparting additional degrees of freedom that enable complex, on-demand deformations. We demonstrate this approach through articulated prototypes capable of executing diverse locomotion strategies, including crawling and rolling, while leveraging on-demand mode switching to perform functional tasks such as cargo transportation, stirring and release. A critical advantage is that all locomotion and mode transitions are driven by a single, uniform magnetic field, thereby decoupling control from function and simplifying the system architecture. The robustness of the mechanism is confirmed through mathematical simulations and a dimensionless analysis that validates its feasibility across scales. The platform’s versatility is ultimately demonstrated by its high-dexterity navigation through a tortuous vascular phantom and its successful execution of targeted cargo delivery within an ex vivo porcine stomach model. This work establishes a readily controllable and scalable framework for designing multifunctional magnetic robots, paving the way for high-precision operations within complex biological environments.

## Introduction

Untethered magnetic actuators hold important potential for minimally invasive medicine [1–4], enabling targeted drug delivery [5,6], remote surgery [7,8], and in vivo diagnostics [9,10]. However, realizing this potential is constrained by a fundamental design challenge: achieving versatile, adaptive functionality without compromising reliable and simple control [11–13]. Operating in complex, unstructured biological environments necessitates an agent capable of on-demand morphological adaptation to switch between distinct locomotion modes and manipulation tasks. This requirement places the field at a critical juncture between 2 dominant yet divergent paradigms, each with inherent limitations. Rigid magnetic actuators [14–17] offer excellent mechanical robustness and predictable locomotion under simple magnetic fields. Yet, their fixed geometries and magnetization profiles intrinsically limit them to single, predefined functions [18–22], lacking the adaptability required for multimodal operations. Conversely, soft magnetic actuators [23–25] can achieve complex, reconfigurable motions through continuous body deformation [26–28]. This shape-morphing capability [29], however, typically incurs costs such as reduced mechanical load capacity [30,31], intricate control demands arising from theoretically infinite degrees of freedom [32–34], and compromised operational stability.

Consequently, this prevailing trade-off has largely confined designs to being either simple and controllable [35–37], or versatile yet complex to actuate [38–40]. Recent efforts to bridge this gap have explored hybrid designs using multimaterial composites or stimuli-responsive polymers to modulate stiffness dynamically [41,42]. While promising, these approaches often face challenges including slow response kinetics, complex fabrication processes that hinder miniaturization, and limited control precision. Thus, a core question remains unresolved: how to engineer a system that embodies the deterministic control and robustness of rigid actuators while retaining the adaptable reconfigurability of soft ones.

Here, we overcome this limitation by introducing an articulated design paradigm based on revolute joints. This strategy bridges the dichotomy by utilizing discrete mechanical articulation to integrate the strengths of both rigid and soft systems. By strategically interconnecting rigid modules, we introduce discrete degrees of freedom that enable complex, on-demand reconfigurations, akin to the versatility of soft robots. Crucially, this is achieved while preserving the essential mechanical robustness and predictable kinematics of the constituent rigid modules. This modular approach not only unlocks multimodal functionality but also provides a framework for assembling a family of specialized actuators from common components.

We demonstrate the efficacy of this paradigm with 4 distinct articulated actuators capable of on-demand modality switching. We show that these robots can perform complex sequences of locomotion and function—such as targeted cargo capture and on-site release—all actuated by a single, uniform magnetic field, thus decoupling spatial control from functional execution. The design’s viability is rigorously established through mathematical simulations of the joint mechanism and dimensionless analysis confirming its cross-scale feasibility. Finally, we validate its real-world potential through cross-scale experiments, demonstrating controlled navigation in a micro-scale simulated blood vessel and precise cargo delivery on a macro-scale ex vivo porcine stomach model. This work establishes a versatile and scalable framework for creating the next generation of high-precision, multifunctional robots for complex biomedical operations.

## Materials and Methods

### Fabrication of magnetic components

A magnetic composite was synthesized by blending NdFeB microparticles (LW-BA 16-7A, XinNuoDe) into a polydimethylsiloxane matrix (SYLGARD 184, Dow Corning, with 10 wt% curing agent). This mixture was spin-coated onto a 3-inch silicon wafer (JingXin) and cured at 80 °C to form a solid film. Cylindrical magnetic pixels were then mechanically punched from the film in 3 dimensions ($D_{1}\;$ = 0.5 mm, $H_{1}$ = 0.55 mm; $D_{2}\;$ = 0.35 mm, $H_{2}\;$ = 0.45 mm; $D_{3}$ = 1.0 mm,$\;H_{3}\;$ = 0.55 mm). A custom electromagnet (WD-50, YP Magnetic) was used to magnetize the pixels with a 1.5-T flux density along either their radial or normal axes, according to the specific design requirements.

### Magnetic actuation

The magnetic actuation and control platform consisted of a square 6-coil Helmholtz electromagnetic system (HuNan YangYi Technology) powered by PSA6004-3-pro power supplies (GuangZhou ZhiYuan Instruments). This integrated system, commanded by a custom Python-based graphical user interface, produced the uniform magnetic fields required for driving the actuators.

### Magnetically driven dynamics

Experiments were performed within a uniform magnetic field (0 to 25 mT) generated across a 100 mm × 100 mm × 50 mm workspace by a custom square Helmholtz hexa-coil system, powered by a programmable power supply.

### Actuators assembly

The nonmagnetic functional modules were designed in Siemens NX 12.0 and fabricated from a biocompatible photopolymer resin using a high-resolution 3-dimensional (3D) printer (microArch S230, Boston Micro Fabrication). Printing was conducted at a layer thickness of 20 μm to ensure high fidelity. Following printing, the modules underwent a post-processing sequence: they were first cleaned with 75% ethanol to remove residual resin, then dried in an oven at 40 to 60 °C, and finally cured for 15 s under ultraviolet light to achieve full mechanical properties. The final actuators were assembled by integrating the pre-magnetized pixels into the 3D-printed modules via an interference fit.

### Fundamental magnetic responses of miniature units

They were characterized using radially magnetized cylinders (0.5 mm inner diameter × 1 mm length) with embedded magnetic discs. When encapsulated within elastomeric tubing (2 mm inner diameter, 0.5 mm wall thickness), systematic field sweeps revealed distinct response maxima and critical thresholds (Figs. S1 to S3) under precisely incremented conditions (frequency steps: 1 Hz; flux density steps: 0.1 mT).

### Statistical analysis

All statistical analyses were performed using Python (version 3.8) with the SciPy and statsmodels libraries. Data are presented as mean ± standard deviation, unless otherwise stated. For each experiment, the sample size (*N*) represents the number of independent replicates per condition, as detailed in the figure captions or main text. Significance was defined as *P* < 0.05.

## Results and Discussion

### Articulated magnetic actuation: A unified framework for versatile biomedical functions

**Fig. 1.**
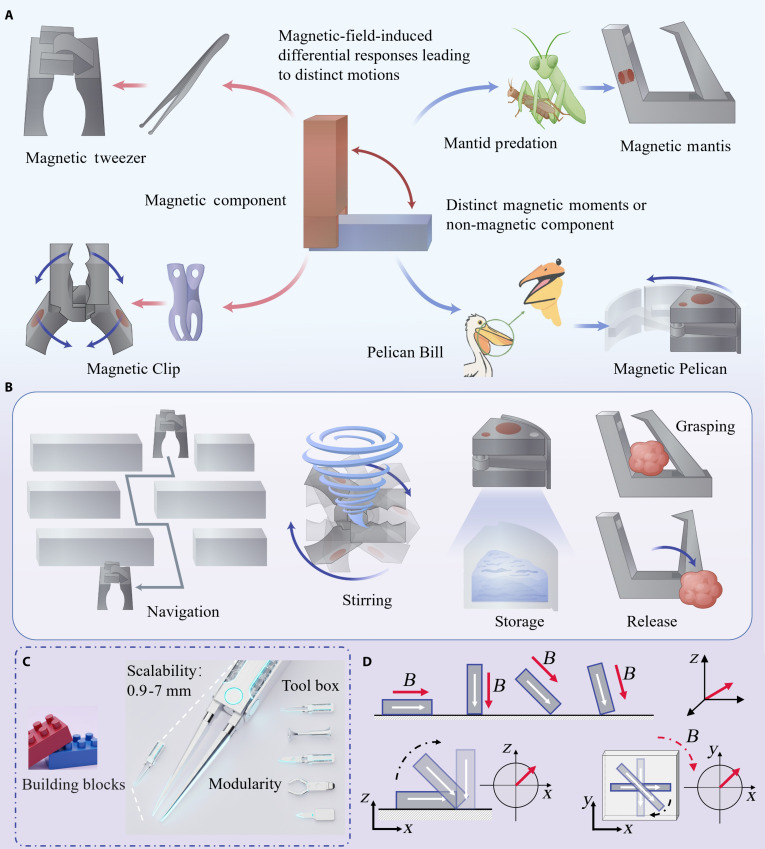
Design paradigm and key features of articulated magnetic actuators. (A) Schematic of the revolute joint principle enabling a family of modular actuators. (B) Illustrations of fundamental operational modes, including navigation, stirring, storage, and grasping. (C) Demonstration of the paradigm’s scalability and modularity, showing micro- and macro-scale versions and constituent components. The schematic diagram was generated by artificial intelligence. (D) Schematic of the unified actuation principle, where a single uniform rotating field controls distinct behaviors like erection, rolling, and planar rotation.

Our articulated magnetic actuator paradigm overcomes the trade-off between functional versatility and simple control by introducing revolute joints to interconnect rigid modules. This approach grants discrete degrees of freedom to otherwise monolithic structures, enabling complex reconfigurations while retaining the mechanical robustness of rigid components. The core of this paradigm is the revolute joint, which allows for controlled relative motion between distinct functional units under a uniform magnetic field (Fig. 1A). By strategically combining magnetic and nonmagnetic modules, this principle allows for the creation of a diverse family of actuators from a common set of building blocks. We designed 4 distinct prototypes based on this concept: the Magnetic Mantis (MM), Magnetic Pelican (MP), Magnetic Tweezer (MT), and Magnetic Clip (MC).

This design framework unlocks a suite of fundamental operational modes essential for biomedical tasks, including targeted navigation, on-site stirring, payload storage, and precision grasping and release (Fig. 1B). The modularity and joint-based design are inherently scalable, enabling the fabrication of actuators across a defined “macro-to-meso” scale range—from several millimeters down to submillimeter dimensions—using the same underlying principles (Fig. 1C). This cross-scale capability, which bridges operation in large anatomical cavities to navigation within confined microvasculature, is critically governed by the need to balance device accessibility with therapeutic function, such as adequate drug payload capacity. Our framework thus provides a targeted solution to this multiscale challenge, demonstrating a clear pathway for scaling device architecture while maintaining unified functionality. A key advantage of our approach is the unified actuation principle: a single, simple, uniform rotating magnetic field is sufficient to drive a wide range of distinct and complex behaviors, such as erection for mode switching, rolling locomotion for navigation, and planar rotation for tasks like stirring (Fig. 1D and Figs. S4 and S5). This effectively decouples the control of sophisticated functions from the need for complex, spatially varying magnetic fields, representing a substantial step toward practical implementation.

### Fabrication and theoretical analysis of magnetically coupled hinged system

**Fig. 2.**
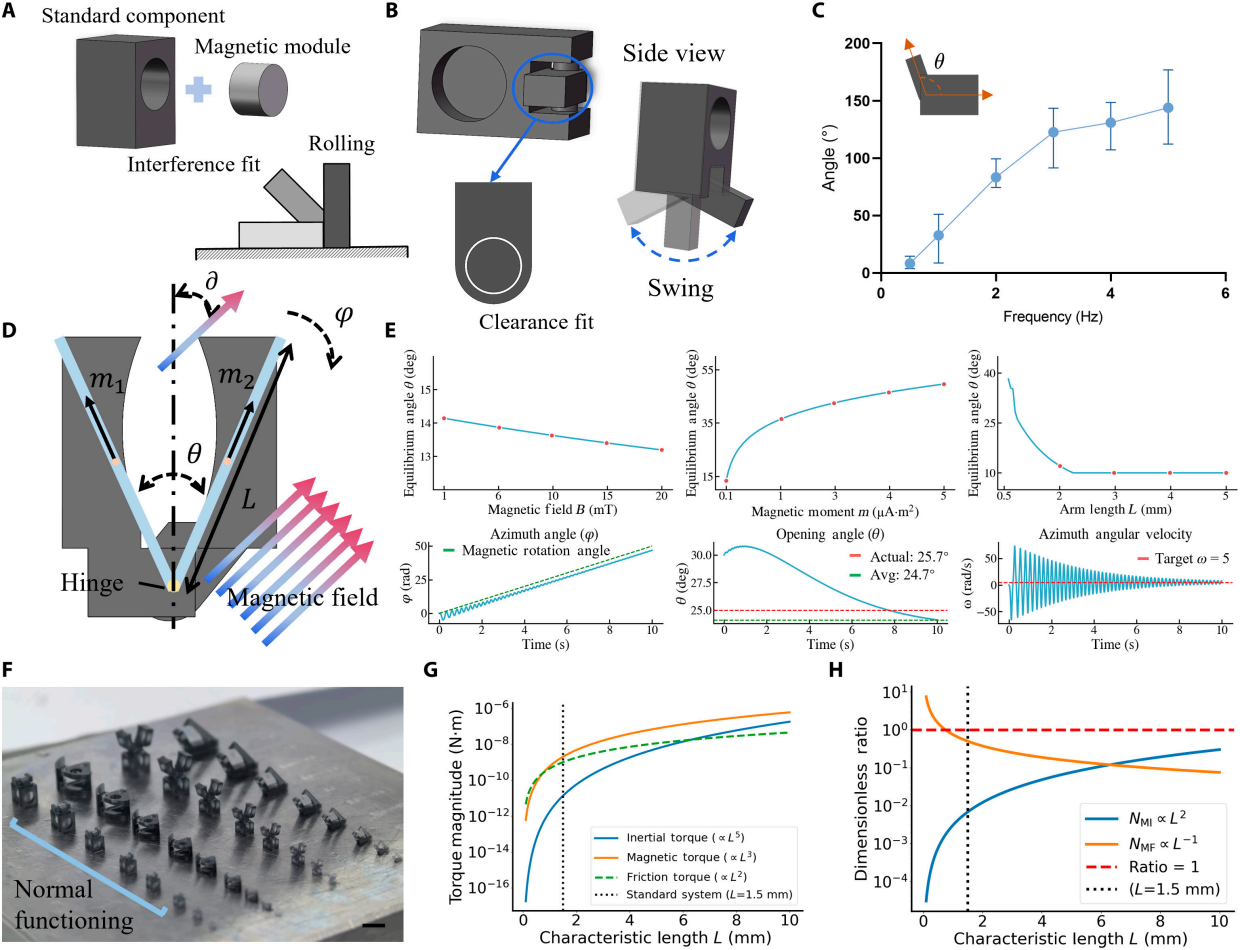
Design, characterization, and theoretical analysis of the articulated joint. (A) Schematic of the interference-fit assembly process for integrating magnetic and nonmagnetic components. (B) Illustration of the revolute joint design and its integration with standard structural parts. (C) Experimental measurement of the joint’s dynamic response, showing the time-resolved opening angle at different driving frequencies. (D) Parameters used for the mathematical model of the articulated magnetic actuators. (E) Theoretical predictions of the joint's static and dynamic behavior. The top 3 panels depict static analyses, showing the equilibrium opening angle as a function of magnetic field *B*, magnetic moment *m*, and arm length *L*, while the bottom 3 panels present dynamic analyses with time-resolved plots of the evolution of azimuth angle, opening angle, and azimuth angular velocity. (F) Fabricated prototypes of the 4 actuator designs at various sizes. Scale bar, 2 mm. (G) Analysis of the dominant magnetic and viscous torques as a function of actuator size. (H) The dimensionless ratio plotted against actuator size. the error bars represent the standard deviation for 5 independent measurements.

The physical realization of our articulated paradigm hinges on a robust and scalable fabrication strategy. We developed a method centered on interference-fit assembly, where pre-magnetized magnetic modules are precisely press-fitted into cavities within 3D-printed, nonmagnetic structural bodies (Fig. 2A). This hybrid manufacturing approach is crucial, as it allows for the independent optimization of both the magnetic and structural components. We can select high-performance magnetic materials for powerful actuation while simultaneously using biocompatible, engineering-grade polymers for the main chassis, a flexibility not easily achieved with monolithic fabrication techniques. The cornerstone of our design is the composite hinge structure that integrates these magnetic and nonmagnetic parts (Fig. 2B and Fig. S6). This design intentionally creates a magneto-mechanical heterogeneity within the actuator. It is this engineered heterogeneity that enables the critical shift from simple, whole-body locomotion to complex, internal functional manipulation. By creating a component that actively responds to the field and another that acts as a passive mechanical linkage, we establish a localized torque gradient under a uniform magnetic field. This internal gradient is the fundamental mechanism that drives all the advanced functionalities, such as grasping and release.

The actuation mechanism relies on the differential response of these components to a uniform rotating magnetic field: the magnetic modules experience a torque that drives their alignment with the field, while the nonmagnetic joint provides a passive mechanical linkage. This creates a controlled torque at the joint, inducing stable, oscillatory opening and closing motions. To characterize its dynamic response, we subjected the joint to a uniform rotating magnetic field, observing large-angle oscillations that are highly dependent on the driving frequency (Fig. 2C). Specifically, at low frequencies, the joint exhibits a stable, coordinated oscillation with minimal phase lag relative to the driving field. As the frequency increases, both the amplitude of the angular oscillation and the phase lag become importantly more pronounced. This complex, frequency-dependent behavior highlights the rich dynamics of the system and necessitates the development of a quantitative model to fully understand and predict its performance.

To quantitatively understand the joint’s dynamics, we developed a comprehensive mathematical model based on the Euler–Lagrange framework. The system’s configuration is fully described by 3 generalized coordinates: the system’s magnetic moment making an angle ∂ with the field, the global azimuth angle φ, and the internal opening angle θ (Fig. 2D). The behavior is governed by a balance of key torques. The internal magnetic torque $\tau_{\text{magnetic}}$, which drives the closing or opening of the joint, arises from the potential energy of the dipole–dipole interaction $U_{\mathrm{int}}$ (Note S1):

τmagnetic=−dUintdθ=−dμ0m24πLsinθ/22+ϵ23/21+sin2θ2dθ (1)

where $U_{\mathrm{int}}\left(\theta \right)$ accounts for the magnetic moments and the geometry of the hinge. The hinge’s motion is opposed by a frictional torque $\tau_{\text{friction}}=-\tau_{k}\text{tanh}\left(\frac{\dot{\theta }}{\omega_{\text{smooth}}}\right)$, which we model using a smoothed smooth Coulomb friction to capture the transition from static to kinetic friction. The external magnetic field $B_{0}$ provides the driving torque $\tau_{\text{external}}=-mB_{0}\text{sin}\left(\partial \right)\;$ that actuates the system. Under quasi-static conditions, the system reaches an equilibrium opening angle θ when all torques on this coordinate balance:

τmagnetic+τexternal+τfriction=0 (2)

Solving this equation yields the theoretical prediction for the joint’s static opening angle as a function of system parameters, which is validated by our experimental results (Fig. 2E and Note S2). To capture the complete time-dependent dynamics observed in experiments, we must also consider inertial effects and viscous drag from the surrounding fluid. The influence of complex bio-fluid rheology, including shear-thinning and yield-stress behaviors, has been incorporated into the theoretical discussion of the system’s dynamics. While numerical simulations utilize a Newtonian model for parametric clarity, the experimental validation conducted in ex vivo biological environments (e.g., on porcine stomach surfaces) confirms that the magnetic actuation delivers sufficient torque to overcome the nonlinear viscous and viscoelastic resistance characteristic of real biological media (Note S3).

The full motion of the system is governed by a set of coupled second-order differential equations (Note S4), which can be expressed in the compact matrix form:

I·q¨+Cqq˙=τext (3)

23marmL20016marmL2ϕ¨θ¨=−2mB0cosθ2sinωBt−ϕ−γϕϕ˙mB0sinθ2cosωBt−ϕ−dUintdθ−τktanhθ˙ωsmooth (4)

where q=(ϕ,θ) is the vector of generalized coordinates and ***I*** is the system’s inertial matrix. ***C*** is a vector containing the dissipative terms (friction and drag), and $\tau_{\mathrm{ext}}$ is the vector of generalized external torques. $m_{\mathrm{arm}}$ is the mass of a single rigid arm. The matrix equation above presents the standard Newtonian form (*n* = 1). For non-Newtonian biological fluids (n≠1), the term $-\;\gamma_{\phi }\dot{\phi }$ is replaced by the generalized nonlinear term $-\;k|\dot{\phi }{|}^{n-1}\dot{\phi }$. Our numerical analysis indicates that for shear-thinning bio-fluids (*n* < 1), the resistive torque at high speeds is reduced compared to the Newtonian baseline, suggesting that the linear model serves as a conservative design benchmark. We solve this system of equations numerically to obtain the time evolution of *φ*(*t*) and *θ*(*t*), which accurately reproduces the dynamic oscillatory behavior seen in Fig. 2E.

Furthermore, the dynamic model provides a powerful framework for analyzing the system’s scalability. A dimensional analysis (Note S5) of the governing equations reveals how the dominant torques scale with the actuator’s characteristic length *L*. As shown in Fig. 2G, the magnetic torques scale as *L*^3^, while the inertial torque scales much more rapidly as *L*^5^, and the frictional torque as $L^{2}$. To understand the interplay between these competing effects, we defined 2 key dimensionless ratios: the magnetic-inertial number $N_{\mathrm{MI}}$, which compares inertial to magnetic forces, and the magnetic-friction number $N_{\mathrm{MF}}$, which compares friction to magnetic forces. Our analysis shows that

NMI=τinertialτexternal∝ρωB2L5M0B0L3=ρωB2M0B0L2 (5)

NMF=τfrictionτexternal∝L2M0B0L3=τkM0B0L−1 (6)

This result has critical implications for actuator design, as plotted in Fig. 2H. The rapid diminishment of inertia confirms that as the device is scaled down, its dynamics become overdamped and characteristic of low-Reynolds-number regimes, leading to highly predictable responses. Conversely, the increasing relative importance of friction highlights that surface forces remain a factor in micro-actuator performance. This theoretical validation, supported by our fabricated multiscale prototypes (Fig. 2F and Fig. S7), confirms the robust, cross-scale viability of our articulated design paradigm.

### Characterization, manipulation, and modularity of the magnetic tweezer

**Fig. 3.**
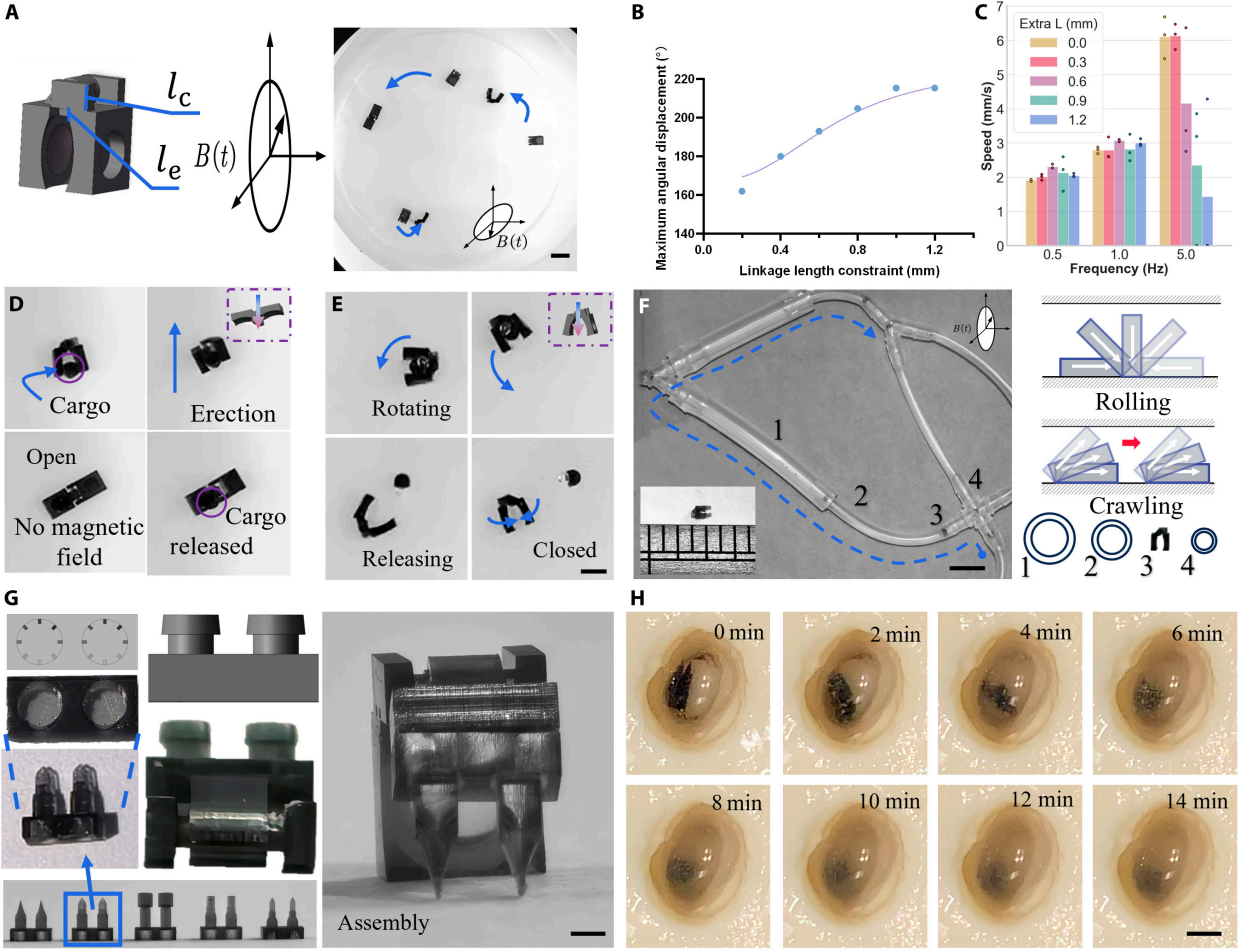
The Magnetic Tweezer (MT): characterization, manipulation, and modularity. (A) Design, magnetization configuration with different responses, and actuation mechanism of the MT. Scale bar, 3 mm. (B) Maximum opening angle as a function of the linkage length constraint. (C) Average locomotion velocity for different extra lengths. (D and E) Programmable open mechanisms: Static magnetic torque opening (D) and dynamic rotational release (E). (F) Navigation through a soft tube of varying diameter and schematics illustrate the 2 observed locomotion modes: normal rolling in wider sections and a crawling motion in sections where the tube is confined. Scale bar, 5 mm. (G and H) Modular customization. (G) Components for modular assembly, showing the main chassis, a library of interchangeable end-effectors, a close-up of the connection mechanism, and an example of a fully assembled custom actuator. Scale bar, 0.5 mm. (H) Demonstration of a scraping task, where a blade-equipped MT performs repeated scraping motion on the tofu block causes the surrounding environment to become murky. Scale bar, 3 mm.

The MT introduces a symmetric, “master–master” architecture, a design choice that fundamentally enhances manipulative capabilities (Note S6). In this configuration, both arms function as active magnetic modules, enabling precise and cooperative control over gripping and release actions under a simple, uniform external magnetic field. As illustrated in Fig. 3A, this is achieved by programming distinct magnetization profiles into each arm. This bilateral actuation ensures that the internal torques generated are balanced and responsive, allowing for fine-tuned control over the tweezer’s opening and closing kinematics, a level of control difficult to achieve in asymmetric master–slave systems. However, the effectiveness of this design depends critically on precise magnetic moment programming; insufficiently configured magnetization can lead to remanent moments that cause partial opening upon field removal, resulting in grip instability and functional failure (Fig. S8).

To optimize the MT’s performance (Note S7), we systematically investigated 2 independent geometric parameters: the linkage length $l_{c}$ and the extra arm length $l_{e}$. First, the linkage length $l_{c}$, which mechanically couples the 2 arms, was identified as the primary determinant of the actuator’s manipulative range. Our experimental results reveal that $l_{c}$ directly governs the maximum achievable opening angle, *α* (Fig. 3B). A longer linkage provides a wider pivot base, allowing the arms to swing to a greater angle before collision, thereby expanding the functional workspace for grasping larger objects. This characterization allows us to tailor the tweezer’s gripping capacity based on specific task requirements.

Separately, we investigated the influence of the extra arm length $l_{e}$, on the actuator’s mobility. $l_{e}$ represents the length of the arm extending beyond the magnetic module, acting as a passive lever. As shown in Fig. 3C, variations in this extra length produce measurable differences in the average locomotion velocity. This effect arises because a longer $l_{e}$ increases the actuator’s effective rolling radius, allowing it to cover more distance per revolution. However, an excessively long, nonactuated arm can also introduce kinematic instability at higher driving frequencies. Therefore, the characterization of $l_{e}$ provides a clear guideline for optimizing the actuator’s speed and stability, complementing the manipulation-focused optimization of $l_{c}$.

The MT’s bilateral design unlocks a repertoire of sophisticated, programmable manipulation tasks that can be triggered by simple, global changes in the external magnetic field. We demonstrate this advanced control by showcasing 2 distinct, on-demand cargo manipulation mechanisms. The first mode, termed static magnetic torque opening (Fig. 3D and Movie S1), exploits the intrinsic magnetic interaction between the gripper arms. During transport, an external magnetic field is applied to maintain the closed gripping state. To actuate the opening, this external field is simply removed (or deactivated). Upon the cessation of the confining external torque, the inherent repulsive magnetic force between the 2 magnetized arms dominates, driving them to snap back to their natural open configuration. As demonstrated, this action effectively disengages the active grasp on the payload, rendering it free from the gripper’s constraint. The second mode, dynamic rotational release (Fig. 3E and Movie S2), utilizes centrifugal effects for cargo separation. In this strategy, the actuator is driven to rotate continuously at high speeds. The resulting centrifugal force, combined with fluid drag, acts on the payload to actively detach it from the gripper. The demonstration also confirms the mechanical capability for in situ grasping (Fig. S9, Note S8, and Movie S3).

To specifically demonstrate the platform’s cross-scale viability (Note S9), we investigated the performance of a miniaturized version of the MT in complex, confined environments that mimic biological conduits. This contrasts with the larger prototypes used for the manipulation tasks described previously. Fig. 3F documents this smaller actuator’s navigation through a soft tube of varying diameter (Movie S4). The accompanying schematics illustrate 2 distinct and observable locomotion modes. In wider sections, the MT employs efficient, high-speed normal rolling. However, where the tube diameter becomes smaller than the actuator’s height, it seamlessly transitions to a slower, inchworm-like crawling motion to guarantee forward progress. This emergent, adaptive behavior is not explicitly programmed into the control field; rather, it arises naturally from the physical interaction between the articulated body and its environment. This demonstrates a form of mechanical intelligence, where the robot’s morphology itself solves a complex navigation problem, underscoring the robustness of the design in unpredictable conditions across different scales. The application of the magnetic tweezers in tortuous microvessels (e.g., arterioles and venules) presents specific hemodynamic challenges. Blood flow imposes a velocity-dependent drag force on the tweezers, while the shear-thinning, non-Newtonian nature of blood means the viscous resistance varies with local shear conditions. Navigating this complex environment requires the magnetic actuation system not only to provide sufficient force to overcome typical microvascular flow drag but also to dynamically adapt to asymmetric hydrodynamic perturbations at bends and bifurcations (Note S11).

A key innovation of our design paradigm is its inherent modularity, which transforms a single robotic platform into a customizable, multitool system. We demonstrate this concept with a modular toolkit developed for the MT chassis. Fig. 3G displays the components for this ecosystem: the main robotic body, a library of interchangeable, task-specific end-effectors (tools), and a close-up view of the robust interference-fit connection mechanism that enables rapid, secure assembly. This “plug-and-play” approach advances the creation of versatile robotic agents that can be reconfigured in the field for a wide array of specialized functions, much like a surgeon selecting different instruments (Fig. S10).

To validate the functional potential of this approach, we tasked a customized MT with performing a demanding mechanical operation far beyond simple gripping. By equipping the actuator with a micro-blade from the tool library, we tested its ability to physically modify a surface. The demonstration in Fig. 3H shows the blade-equipped MT executing repeated, high-force scraping motions on a soft tissue phantom (a tofu block). The actuator successfully causes the surrounding environment to become murky, showcasing its capability to generate sufficient force and maintain stability during aggressive, tool-based tasks.

### Functional demonstrations of the magnetic mantis and magnetic pelican

**Fig. 4.**
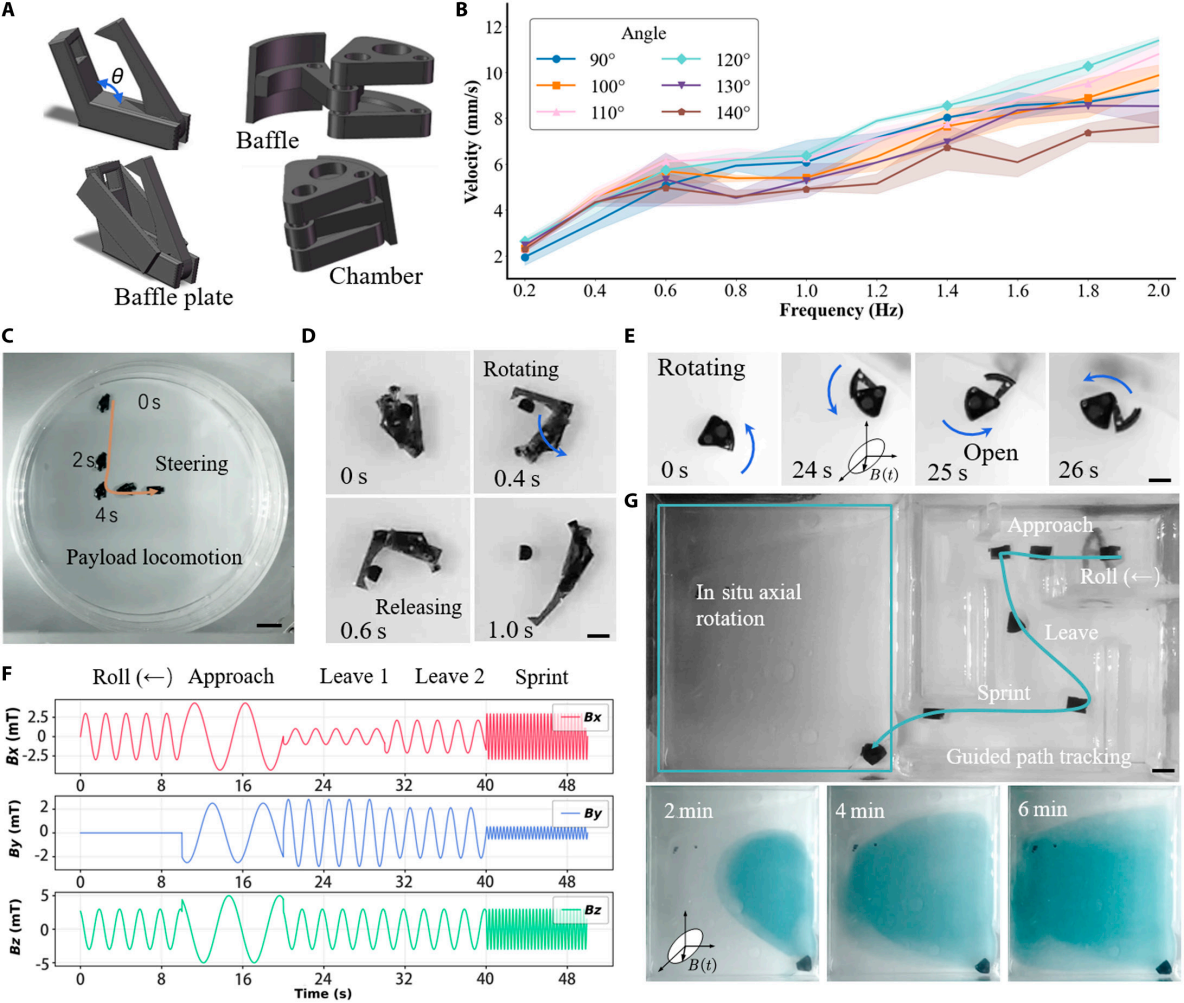
Functional demonstrations of the Magnetic Mantis (MM) and Magnetic Pelican (MP). (A) Schematics of the MM and MP master–slave designs. (B) Locomotion velocity of both actuators versus driving frequency and structural angle. (C) The MM performing loaded cargo transport along a trajectory with a 90° turn. Scale bar, 5 mm. (D) Magnetically triggered mode-switching of the MM for payload release. Scale bar, 1 mm. (E) Magnetically triggered opening of the MP's baffle for cargo deployment. Scale bar, 1 mm. (F) Time sequence of the magnetic flux density used for actuation control. The *Y*-axis labels *B_x_*, *B_y_*, and *B_z_* represent the magnetic field in the *x*, *y*, and *z* directions, respectively. The *X*-axis is time in seconds. (G) The MP shown in a simulated mission: navigation, drug release, and accelerated fluid mixing via on-site rotation. Scale bar, 3 mm. The error bars represent the standard deviation for 3 independent measurements.

Drawing inspiration from biological systems, we developed a class of actuators based on an asymmetric “master–slave” configuration. In this design, a magnetized “master” module actively responds to the external field, driving the motion of a passive, nonmagnetic “slave” module through an articulated joint. Fig. 4A schematically illustrates 2 distinct embodiments of this principle: the MM and the MP. This design leverages asymmetry to create specialized, single-purpose mechanisms like grippers or hatches, making it ideal for robust, task-specific applications. Focusing first on the MM, which is designed for robust gripping, we systematically optimized its performance by investigating the influence of its internal structural angle ($\theta_{s}$). A comprehensive characterization of locomotion velocity was performed as a function of both driving frequency and this key geometric parameter (Fig. 4B). The results reveal that while the structural angle has a negligible effect on velocity at low frequencies, it becomes a critical factor at higher frequencies. Specifically, the data show that a configuration with $\theta_{s}$ = 120° achieves superior velocity above 1 Hz compared to other angles. This finding provides a clear, data-driven design rule for maximizing the MM’s navigational efficiency, especially for high-speed operations. This optimized MM serves as the basis for the functional demonstrations that follow.

Building upon the optimized design, we demonstrate the MM’s efficacy in robust delivery-and-release tasks. As shown in the time-lapse sequence of Fig. 4C, the actuator securely transports a solid cargo along a predefined path, exhibiting exceptional maneuverability and stability. It precisely navigates a sharp 90° turn without losing its grip—a demonstration of the stable frictional engagement maintained by the directionally decoupled magnetic control strategy. The critical on-demand release function is enabled by a magnetic field-based mode switch. Upon reaching the destination, the external field is switched from a uniform *Z*-axis rotation to a high-frequency uniform rotation in the *XY*-plane (Movie S5), triggering immediate payload detachment. This orthogonal control scheme—where locomotion is driven by fields involving the *Z*-axis and release is exclusively activated in the *XY*-plane—constitutes a functionally decoupled master–slave configuration. The spatial separation of the actuation fields ensures that both locomotion and release are robust, predictable, and mutually noninterfering under normal operation.

As illustrated in the schematics and snapshots of Fig. 4D, this specific trigger field induces a large-amplitude pivotal motion in the master module. This motion is directly transmitted to the slave module through the articulated joint, forcing it to swing outwards. This coordinated reconfiguration rapidly and dramatically increases the gripper aperture from a closed state to its maximum opening, ensuring a clean and instantaneous release of the cargo. This seamless integration of high-speed navigation, stable gripping, and triggered release demonstrates a complete and controlled manipulation cycle, validating the MM’s potential for automated, high-throughput tasks in settings like micro-assembly or biological sample handling.

In parallel, the MP demonstrates the versatility of the master–slave concept by adapting it for complex fluidic tasks, specifically the targeted delivery and enhanced mixing of liquid payloads. Like the MM, the MP can be precisely navigated to a target location using a rotating magnetic field. Its innovation lies in the payload deployment mechanism, where a baffle acts as the “slave” module, sealing a liquid-filled cavity. As depicted in Fig. 4E, a switch to an *XY*-plane magnetic field triggers the release (Movie S7). This field exerts a torque on the magnetized “master” component, causing it to rotate and, in turn, retract the connected baffle. This action unseals the cavity, allowing the stored liquid to be released into the ambient environment (Fig. S11).

We demonstrate the system’s multifunctional capabilities through a simulated mission (Movie S7). To mitigate payload leakage during transport, the MP design incorporates integrated internal mechanical baffles within the payload chamber to dampen fluid sloshing. Furthermore, a water-in-oil transport strategy is employed, where the aqueous payload is encapsulated within an immiscible oil phase, enhancing containment under standard operating conditions. Beyond containment, the system’s ability to perform on-demand payload release is governed by the lid’s rotational dynamics, specifically its moment of inertia. To experimentally validate this mechanism, a dedicated high-inertia variant was fabricated by embedding high-density zirconia into the lid. As shown in Fig. S12 and Movie S8, this variant achieves immediate lid opening upon magnetic activation, confirming the fundamental feasibility of rapid, triggered release (Note S10). This mechanistic understanding highlights a key design trade-off: while increased inertia enables fast opening, it also compromises buoyancy balance and locomotion agility. Therefore, for tasks demanding precise navigation—such as maze traversal—a lightweight design is favored. In this configuration, the slower lid rotation effectively operates in a stirring-assisted release mode, enabling gradual payload dispersion without sacrificing mobility. Together, these insights allow the MP system to be strategically tailored, prioritizing either instantaneous release or high maneuverability according to specific operational requirements. Following a pre-programmed sequence of magnetic fields (Fig. 4F), the MP navigates to a target location using the “Rolling Mode”. Upon reaching the target at the water–oil interface, the robot executes a localized, high-speed rolling maneuver in the *XY*-plane (Fig. 4G). This motion actively shears and mixes the payload with the surrounding aqueous fluid, accelerating its dispersion to complete the delivery phase (Fig. S13).

### Functional demonstrations of the magnetic clip

**Fig. 5.**
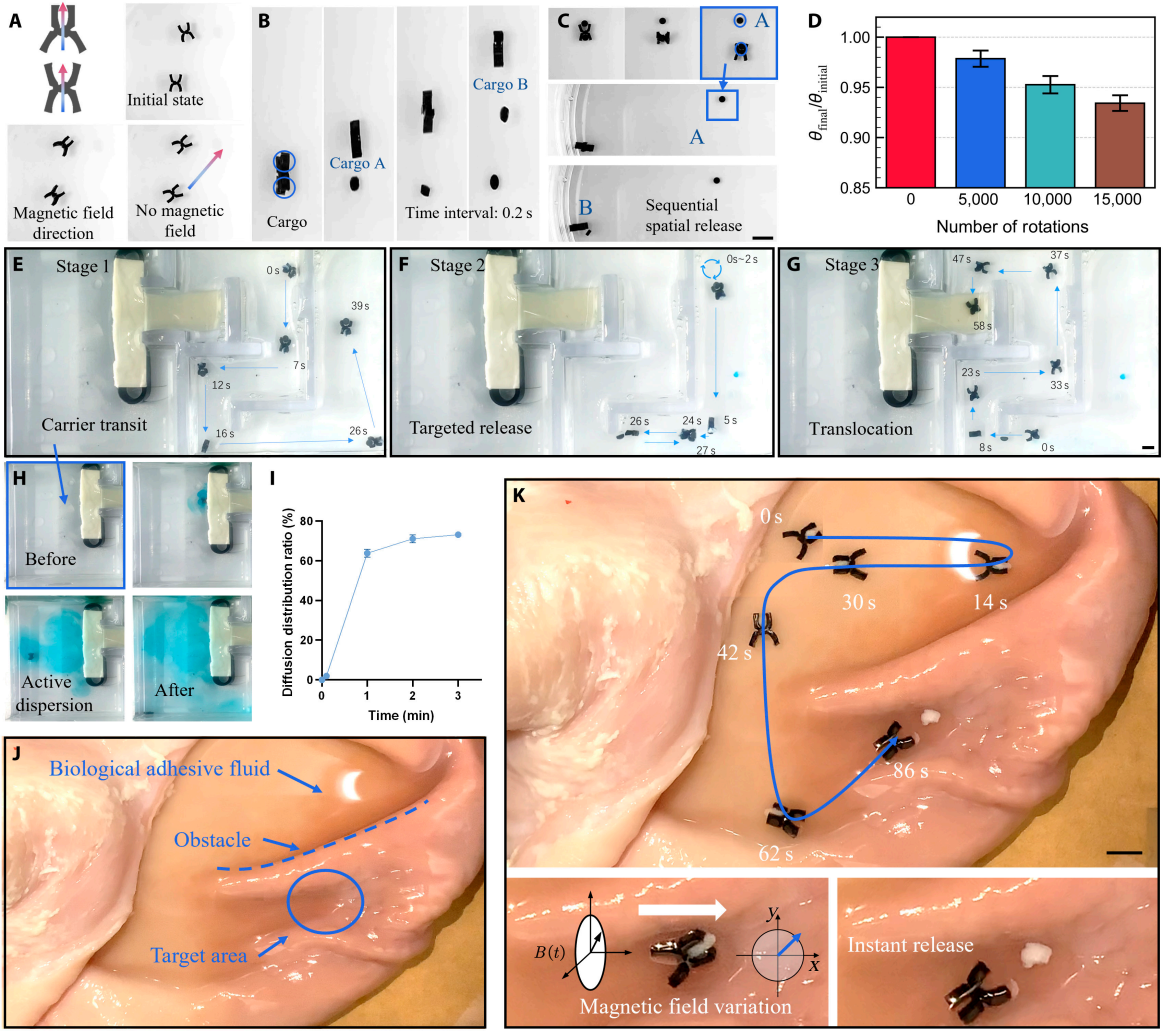
The Magnetic Clip (MC): programmable actuation, multistage tasks, and ex vivo demonstration. (A) Distinct static configurations of 2 differently magnetized MCs under the same uniform field. (B) Near-simultaneous release of 2 payloads shown in a 0.2-s image sequence. (C) Sequential payload delivery to 2 distinct target locations. Scale bar, 5 mm. (D) Mechanical fatigue test demonstrating the actuator's durability over numerous actuation cycles. The normalized retention of the gripper’s opening angle, defined as the ratio of the angle measured after a specified number of actuation cycles ($\theta_{\text{final}}$) to the initial, pre-fatigue angle ($\theta_{\text{initial}}$). This metric, expressed as $\theta_{\text{final}}/\theta_{\text{initial}}$, quantifies the actuator’s mechanical durability and its resistance to performance degradation over extended use. (E to G) Image sequence of a multistage mission in a maze: (E) solid cargo transport, (F) release at a first target, and (G) liquid cargo release and stirring at a second target. Scale bar, 5 mm. (H) Visualization of enhanced fluid mixing after stirring. (I) Plot of normalized diffusion area versus time, quantifying mixing performance. (J) A pig stomach environment filled with biological adhesive fluid and obstacles. (K) MC performing targeted cargo delivery on the surface of an ex vivo pig stomach. Scale bar, 5 mm. The error bars represent the standard deviation for 3 independent measurements.

The MC epitomizes the functional complexity achievable with our design paradigm, leveraging magnetization-programmed responses to unlock multistage, logic-based operations. The core innovation is that the MC’s behavior is dictated not only by the external field but also by its own pre-programmed internal magnetic signature. This principle is strikingly illustrated in Fig. 5A, where 2 geometrically identical MCs, distinguished only by their internal magnetization profiles, exhibit opposing behaviors—one snapping closed while the other remains open—within the exact same uniform magnetic field. This effectively encodes a unique logical state directly into the material’s physical structure. To enable the MC to reliably execute such demanding and intricate tasks, we first confirmed its mechanical robustness. A rigorous fatigue test (Fig. 5D) subjected the articulated joint to thousands of actuation cycles. The results show no evidence of structural degradation or performance decay, affirming the MC’s durability for long-term, repetitive operations. Building on this robust and programmable foundation, we demonstrate the MC’s capacity for sophisticated payload deployment through 2 vastly different, pre-programmable release strategies. First, we showcase a “burst release” strategy for rapid, area-of-effect delivery. As seen in Fig. 5B, a specific magnetic field sequence triggers the near-simultaneous ejection of 2 distinct payloads in under 0.2 s (Movie S9). Conversely, demonstrating fine-grained spatiotemporal control, we executed a sequential delivery mission (Fig. 5C). In this scenario, the MC first navigates to a target site to release its primary payload. It then retains its secondary payload while traveling to a second, distinct location for a subsequent, targeted release (Movie S10). The MC achieves controlled sequential payload release through a 2-stage strategy employing distinct physical principles. The first stage enables selective detachment of the initial payload via programmed static torque asymmetry. Although the clip’s mechanical structure is symmetric, the magnetic moments of its 2 arms are intentionally offset. Application of a high-intensity magnetic field in a specific direction generates a strong opening torque on the designated “target arm”, reducing its contact force and static friction to release the first payload. Simultaneously, the field exerts a closing or neutral torque on the opposite “safe arm”, retaining the second payload. After the first delivery, the system switches to a high-frequency rotating magnetic field for the second stage. This actuation spins the entire clip, generating centrifugal force that exceeds the residual grip strength of the second arm, thereby ejecting the remaining payload (Fig. S14). The ability to execute these fundamentally different missions—from a single explosive burst to a meticulous, multilocation delivery—without any change to the actuator’s physical design is a hallmark of this approach. Mechanical fatigue tests were conducted, and as illustrated in Fig. 5D, the robot’s aperture maintained normal opening and closing functionality after 15,000 cycles.

To culminate our demonstrations, we programmed the MC to execute a single, continuous mission that synergistically integrates its diverse capabilities: multimodal cargo handling, precision navigation, and active fluid manipulation. We designed a complex maze to serve as a challenging testbed for this multistage delivery task. The mission commences with the MC deftly navigating the tortuous channels while securing a solid payload (Fig. 5E, Fig. S15, and Movie S11). Upon reaching the first pre-defined target, it is commanded to precisely deposit the solid cargo (Fig. 5F). Without reset or retrieval, the mission seamlessly transitions to its next phase. The MC proceeds to a second, distinct target site where it executes a fundamentally different, pre-programmed function: releasing a liquid payload. Crucially, it then initiates on-site spinning to act as a dynamic micromixer (Fig. 5G). The profound effectiveness of this active mixing is confirmed both visually through dye dispersion (Fig. 5H) and quantitatively by the rapid expansion of the normalized diffusion area over time (Fig. 5I), which dramatically outpaces passive diffusion. This complex, sequential operation validates the MC as a versatile robotic agent capable of executing a pre-programmed “script” of diverse actions.

Quantitative characterization under controlled stationary fluid conditions confirms the precise and responsive control of the MC (Figs. S16 and S17 and Movie S12). Trajectory-following experiments over multiple cycles yield a root-mean-square tracking error of ~2.0 mm (<0.3 body lengths, BL = 7 mm), demonstrating robust locomotion control. The accuracy of the dynamic tumbling release mechanism was evaluated over multiple trials, achieving an average payload placement within ~1.0 cm (~1.4 BL) of the target center. The actuation system responds to commands with millisecond-level latency, with the robot’s motion being primarily governed by fluid dynamics rather than control delay. These results verify the system’s intrinsic capability for precise navigation and targeted release.

Finally, to bridge the gap between idealized laboratory conditions and clinically relevant scenarios, we assessed the platform’s performance in a challenging ex vivo biological environment. We evaluated the MC’s performance on the interior surface of an ex vivo porcine stomach, a biological environment characterized by mucus coverage, tissue folds, and physical obstructions (Fig. 5J). Despite these challenges, the MC successfully navigated from a starting point to a predefined target site. As shown by its motion trajectory (Fig. 5K and Movie S13), the device reached the destination and executed a controlled release of its cargo. To assess mobility in unstructured biological environments, the robot was tested on a biomimetic complex terrain made of crushed tofu within a biphasic fluid medium, simulating the mechanical properties of soft tissue and luminal contents. As shown in Movie S14, the MC successfully traversed this irregular landscape, climbing soft ridges and navigating depressions via tumbling locomotion. This result confirms that the actuation provides sufficient torque and traction for effective motion on compliant, slippery, and obstacle-rich substrates, supporting its potential utility in realistic anatomical settings. This demonstration of targeted locomotion and delivery in a complex, anatomically relevant model indicates that the articulated design can operate effectively in unstructured, slippery environments, supporting its potential for future biomedical applications.

## Conclusion

In summary, this work addresses a fundamental dilemma in magnetic actuators: the trade-off between the simple control of rigid robots and the functional versatility of soft robots. We introduce and validate a novel design paradigm based on articulated revolute joints that synergistically combines the strengths of both systems. By strategically interconnecting rigid magnetic and nonmagnetic modules, our approach creates discrete degrees of freedom, enabling complex, on-demand reconfigurations while retaining mechanical robustness and predictable kinematics.

Leveraging this modular framework, we developed a family of distinct actuators capable of sophisticated, multistage tasks—including targeted navigation, programmable cargo release, and active fluid mixing—all driven by a single, uniform magnetic field. The platform’s robustness and cross-scale viability were confirmed through mathematical modeling, dimensionless analysis, and successful demonstrations ranging from navigation in micro-scale channels to targeted delivery on a macro-scale ex vivo porcine stomach. Crucially, this design paradigm establishes a modular toolkit, enabling the rapid assembly of a diverse family of task-specific actuators from a common set of components. The versatility of our approach was further underscored by the creation of a toolkit for the MM.

Critically, our approach decouples complex functional execution from the need for complex control fields, establishing a scalable and readily implementable framework for creating the next generation of multifunctional medical actuators. This work paves the way for high-precision robotic agents capable of performing complex operations in challenging biological environments, offering strong potential for applications in targeted drug delivery, minimally invasive surgery, and diagnostics.

## Data Availability

All data needed to evaluate the conclusions in the paper are present in the paper and/or the Supplementary Materials. Additional data related to this paper may be requested from the corresponding author upon reasonable request.

## Supplementary Materials

Supplementary 1 Notes S1 to S11 Figs. S1 to S18 Movies S1 to S14
