# Supplementary material for: Articulated Untethered Magnetic Actuators for Multimodal and Cross-Scale Operations
Source: Cyborg Bionic Syst. 2026 May 19;7:0560. doi: 10.34133/cbsystems.0560 (PMC13184954; doi:10.34133/cbsystems.0560)
Supplement: Supplementary 1 — Notes S1 to S11 Figs. S1 to S18 Movies S1 to S14 [file cbsystems.0560.f1.zip › Supporting Information0310.docx]

Supporting Information

Articulated Untethered Magnetic Actuators for Multimodal and Cross-Scale Operations

Title

Articulated Untethered Magnetic Actuators for Multimodal and Cross-Scale Operations

**Authors**

Zhixian Chen1, †, Xiaoyu Zhao1, †, Ying Liu1 and Shengli Mi1, 2, *

**Affiliations**

1 Bio-manufacturing Engineering Laboratory, Institute of Data and Information, Tsinghua Shenzhen International Graduate School, Tsinghua University, Shenzhen, Guangdong 518000, China

2 Center of Double Helix, Tsinghua Shenzhen International Graduate School, Tsinghua University, Shenzhen, Guangdong 518000, China

*Address correspondence to: [mi.shengli@sz.tsinghua.edu.cn](mailto:mi.shengli@sz.tsinghua.edu.cn)

†These authors contributed equally to this work.

Supporting Notes

**Note S1.** Analytical solution for the interaction energy between two magnetic dipoles.

The interaction energy between two magnetic dipoles is given by the general expression:

Where is the vacuum permeability, and are the magnetic moment vectors, is the separation distance, and is the unit vector along the direction connecting the two dipoles. Consider two identical magnetic dipoles with magnetic moment magnitude , each located at the midpoint of rigid arms of length connected by a hinge. The opening angle between the arms is . The magnetic moments are oriented along their respective arms.

The magnetic moment vectors in Cartesian coordinates are:

The unit displacement vector from to is:

The separation distance between the dipoles is:

Compute the necessary dot products:

Substitute the dot products into the dipole interaction energy formula:

Substitute the simplified trigonometric expression to obtain the final result:

**Note S2.** Theoretical analysis of magnetically coupled hinged system.

When a magnetic dipole with moment is placed in a uniform external magnetic field , it experiences a torque. This torque acts as a restoring force, tending to align the dipole moment with the field direction. The magnitude of this torque is maximum when the dipole is perpendicular to the field ( ) and zero when it is aligned or anti-aligned (  or ).

Where is the angle between the magnetic moment vector and the external magnetic field vector. When subjected to an external magnetic field B₀ aligned with the system's bisector, the total opening angle θ is symmetrically divided. Consequently, the angle of orientation for each arm relative to the field is θ/2.

The potential energy arising from the magnetic interaction between the two dipoles is central to the system's behavior. For two identical dipoles separated by a distance  with a relative orientation θ, the system will naturally tend to move towards a configuration that minimizes this potential energy.

Where the is the vacuum permeability. The separation distance is between the centers of the two magnets, related to the arm length   and opening angle   by  .

A computational singularity occurs as  because , leading to a divergence in energy. To circumvent this unphysical behavior and ensure numerical stability, a softening parameter  is introduced, representing a minimum effective separation. The softened distance and modified potential energy become:

The internal magnetic torque, derived as the negative gradient of the internal potential energy with respect to the angle , is given by:

Due to the complexity of the analytical derivative, a central difference approximation is employed for numerical computation:

where the step size  is chosen adaptively to maintain precision:

Hinge friction is modeled using a smoothed smooth Coulomb friction model, which provides a continuous and differentiable function that captures the transition from static to kinetic friction.

Here is the angular velocity ( ). is the magnitude of the kinetic friction torque and is a small angular velocity constant that controls the smoothness of the transition region near  .

Under quasi-static conditions, the system reaches equilibrium when the net torque acting upon it is zero. It states that for the system to be in equilibrium, the sum of all torques—internal magnetic, external magnetic, and frictional—must be zero. Solving this equation for  yields the equilibrium angles of the hinged structure.

**Note S3.** Viscous Drag and Biological Fluid Considerations.
The primary dissipative force acting on the robot is fluid drag. In a simplified Newtonian environment (e.g., water or saline), this can be modeled as a linear viscous drag acting on the coordinate:

where  is a constant viscous drag coefficient dependent on the fluid viscosity  and geometry.

However, in realistic biological environments (e.g., mucus, blood, gastric fluids), the fluid exhibits complex non-Newtonian properties, such as shear-thinning behavior and viscoelasticity. To account for these effects, the drag torque is more accurately described by a generalized non-linear model:

For a shear-thinning fluid (e.g., gastric mucus), the effective viscosity  decreases with increasing shear rate. This can be approximated using a Power Law (Ostwald-de Waele) model:

where  is the flow consistency index and  represents the shear-thinning behavior. Consequently, at higher rotational speeds (high ), the effective drag experienced by the robot in biological fluids may be lower than predicted by a linear model, potentially enhancing mobility. Conversely, at low speeds or static conditions, the presence of a yield stress (Bingham plastic behavior) in mucus may introduce an additional resistive threshold:

While the simplified linear model () used in our simulation provides a fundamental understanding of the dynamics, the actual performance in bio-fluids will vary dynamically with actuation frequency. Our ex-vivo experiments (Movie S10) inherently incorporate these non-linear effects, demonstrating that the magnetic torque provided by the actuator is sufficient to overcome the complex rheological resistance of real biological fluids.

**Note S4.** A system of coupled second-order differential equations that govern the complete motion dynamics of the magnetically coupled hinged structure.

The system's configuration is fully described by two generalized coordinates: the azimuth angle  and the opening angle .The dynamics of the system are governed by the Euler-Lagrange equations, which can be expressed in matrix form as:

Where  is the vector of generalized coordinates. : is the vector of generalized angular accelerations. is the system's inertial matrix.

is the vector of total generalized torques acting on the system. The inertial matrix  relates the angular accelerations to the applied torques. For this system, the off-diagonal terms are zero. The diagonal elements are derived from the system's kinetic energy.

- : The mass of a single rigid arm.
- : The total moment of inertia for rotation about the central z-axis.

It is the sum of the moments of inertia of two rods rotating about one end:

- : The generalized mass corresponding to the opening angle  . Based on a derivation from the system's kinetic energy, this term is:

Thus, the inertial matrix is:

For the symmetric two-arm system with arm mass  and length , the inertial matrix is diagonal:

The torques defined in the static analysis are now applied to the dynamic equations. The external torque, however, must be generalized for the two degrees of freedom under a time-varying field.

For a time-varying externally applied rotating magnetic field, . The potential energy of this interaction is:

which evaluates to:

The generalized torques are the negative gradients of this potential,

A linear drag model representing fluid resistance to the overall rotation, acting on the coordinate:

where   is a viscous drag coefficient.

Combining all components yields the final system of coupled, second-order ordinary differential equations that govern the actuator's motion:

*The matrix equation above presents the standard Newtonian form (). For non-Newtonian biological fluids (), the term  is replaced by the generalized non-linear term . Our numerical analysis indicates that for shear-thinning bio-fluids (), the resistive torque at high speeds is reduced compared to the Newtonian baseline, suggesting that the linear model serves as a conservative design benchmark.*

This system of equations is solved numerically using Runge-Kutta methods to yield the time evolution of the system's state, and .

**Note S5.** Dimensional analysis and scaling laws

For micro-scale systems, such as the one considered ( ), the relative importance of physical forces can differ fundamentally from that in the macroscopic world. A dimensional analysis of the governing equations reveals these crucial scaling laws. To analyze the effect of size, we consider a geometrically self-similar scaling, where all dimensions (length  , width  , height  ) scale proportionally. The key torques in the system scale with the characteristic length  as follows:

**Inertial Torque (** **)**: This torque is proportional to the moment of inertia and the characteristic angular acceleration,

Given that the arm mass  , the inertial torque scales as:

**External Magnetic Torque (** **)**: This driving torque is proportional to the magnetic moment,  . Since the magnetic moment  , the scaling relation is:

**Internal Magnetic Torque (** **)**: This restoring torque arises from dipole-dipole interaction, scaling as

**Frictional Torque (** **)**: frictional torque scale with surface area ( ).

The dynamic model enables investigation of system behavior across different length scales through dimensional analysis. For geometrically self-similar scaling where all dimensions scale proportionally with characteristic length , the key torques exhibit distinct scaling relationships:

**Inertial Torque**:

**External Magnetic Torque**:

**Internal Magnetic Torque**:

**Frictional Torque**:

Magnetic-Inertial Number ( ) quantifies the relative importance of inertial forces to magnetic actuation forces.

The ratio of inertial to magnetic torque scales with the square of the characteristic length ( ). Magnetic-Friction Number ( ) quantifies the relative importance of surface-dominant forces (like static friction) to magnetic actuation forces.

The ratio of frictional to magnetic torque scales with the characteristic length ( ).The derived scaling laws directly address the unique physics of micro-scale systems.
 indicates that friction becomes more significant at small scales, though the increase is linear rather than cubic. This implies that frictional forces remain a critical factor in micro-actuator performance.  demonstrates rapid diminishment of inertial effects, resulting in overdamped dynamics characteristic of low-Reynolds-number regimes. The system exhibits instantaneous response to field changes with minimal overshoot.

**Note S6.** Low frequency synchronous rolling and high-frequency centrifugal/inertial unloading strategy

Synchronous Locomotion under Low-Frequency Actuation:

All rolling locomotion is driven by a magnetic field rotating in a plane that includes the Z-axis (e.g., the YZ-plane), within a strictly confined low-frequency band (). In this regime, the robot responds linearly and synchronously, rotating at an angular velocity ω that matches the field frequency with negligible phase lag. Crucially, the centrifugal force ) generated at these low rotational speeds remains substantially below the magnetic holding force () that secures the payload. This inherent force balance ensures stable transport and prevents any unintended detachment during locomotion.

Triggered Release via Centrifugal Ejection:

In contrast, payload release is a deliberate function, activated only by applying a rotating magnetic field exclusively in the XY-plane. To ensure rapid and reliable ejection, the release protocol actively optimizes the force balance. We exploit the scaling relationships where . While a high frequency is necessary to generate sufficient , an excessively high magnetic flux density (B) can strengthen and hinder release. Therefore, our optimized release command employs a high-frequency field combined with a slightly reduced magnetic field intensity. This modulation ensures the centrifugal force decisively exceeds the magnetic adhesion (), enabling immediate and repeatable payload separation.

Rolling‑Induced Release under Z‑Axis Field:

Alternatively, release can also be triggered while maintaining the magnetic field in a plane containing the Z‑axis. By driving the robot into a back‑and‑forth rolling motion within the same low‑frequency band used for transport, the periodic reversal of motion introduces inertial disturbances and transient reductions in effective magnetic adhesion. This dynamic destabilization—without requiring a change of the field plane—causes the payload to detach when the momentary inertial forces exceed the magnetic holding force. This method provides a simple, plane‑consistent strategy for release where high‑frequency rotation in the XY‑plane is not desirable.

**Note S7.** Classification magnetic field correction and active pose reset mechanism.

To guarantee robust logic-based operation under real-world orientation uncertainties, the system employs a hierarchical recovery strategy that combines passive realignment with active posture resetting. Upon mode transitions, the inherent magnetic interaction generates a restoring torque that rapidly reorients the robot toward the field direction, correcting minor angular deviations through field-following behavior. For significant misalignments such as an inverted landing, an active initialization step is applied: a brief low-frequency rolling field with a π/2 phase shift deterministically reorients the robot into a stable horizontal posture. This alignment is essential because the high-frequency release field is applied exclusively in the XY plane, requiring the robot’s magnetic moment to lie within the excitation plane (Figure S4**)**. Once correctly aligned, the designated actuation field is applied, ensuring reliable execution of the intended logic function. This integrated approach enables the system to recover from a range of landing uncertainties while maintaining operational precision and repeatability.

**Note S8** Active cargo capture and transport using the Magnetic Tweezer (MT).

While our original study focused on the transport of pre‑loaded cargo—reflecting clinical workflows such as chemoembolization—the additional demonstration confirms the mechanical capability for in‑situ grasping. As shown in Figure S9, the MT was placed near a target cargo in an open state; a static magnetic field actuated the arms to securely enclose the cargo, after which a rotating field induced rolling locomotion while maintaining grip. This procedure, conducted via manual teleoperation under visual guidance, validates that the actuation mechanism provides sufficient force for active capture and transport. Future work will focus on implementing closed‑loop control and AI‑assisted navigation to achieve fully autonomous alignment and grasping.

**Note S9.** The cross-scale capability.

The cross-scale capability presented in this work is defined as the fabrication and magnetic actuation of robots across an order of magnitude in size, from the meso-scale (≈ 900 μm) to the macro-scale (≈ 7 mm). This range was engineered to address two distinct clinical environments: large cavity operations and navigation within tortuous microvasculature. Scaling in this context is critically constrained by volume-dependent therapeutic function, not merely linear dimension. The three-order-of-magnitude difference in volume between the two robot scales dictates their drug payload capacity. The 900 μm robot is designed as a functional lower limit, providing sufficient miniaturization for vascular access while retaining a volume capable of encapsulating a clinically meaningful therapeutic dose, thereby balancing the requirements of accessibility and therapeutic function for targeted delivery applications.

**Note S10.** System-Oriented Optimization of Containment and Triggered Release in MP

To mitigate payload leakage during transport, the MP design incorporates integrated internal mechanical baffles within the payload chamber to dampen fluid sloshing. Furthermore, a water-in-oil transport strategy is employed, where the aqueous payload is encapsulated within an immiscible oil phase, enhancing containment under standard operating conditions.

Beyond containment, the system’s ability to perform on‑demand payload release is governed by the lid’s rotational dynamics, specifically its moment of inertia. To experimentally validate this mechanism, a dedicated High‑Inertia Variant was fabricated by embedding high‑density zirconia into the lid. As shown in Figure S7 and Movie S7, this variant achieves immediate lid opening upon magnetic activation, confirming the fundamental feasibility of rapid, triggered release.

This mechanistic understanding highlights a key design trade‑off: while increased inertia enables fast opening, it also compromises buoyancy balance and locomotion agility. Therefore, for tasks demanding precise navigation—such as maze traversal—a Lightweight Design is favored. In this configuration, the slower lid rotation effectively operates in a stirring‑assisted release mode, enabling gradual payload dispersion without sacrificing mobility. Together, these insights allow the MP system to be strategically tailored, prioritizing either instantaneous release or high maneuverability according to specific operational requirements.

**Note S11.** Hemodynamic Considerations for Magnetic Actuator Navigation in Tortuous Microvessels

The effective operation of untethered magnetic actuators within the dynamic environment of living microvessels, such as tortuous arterioles and venules, requires a nuanced understanding of local hemodynamics. Blood in these small-diameter vessels exhibits non-Newtonian, shear-thinning behavior, where its effective viscosity decreases with increasing shear rate. This property creates a direct coupling between local flow velocity and viscous resistance. In regions of low shear, such as near vessel walls or within aneurysmal expansions, the higher apparent viscosity presents a significant resistive torque against actuator motion. Conversely, in high-shear regions like the vessel center or stenotic segments, viscosity drops toward a Newtonian plateau, but the fluidic drag force imposed by the flow velocity itself becomes the dominant resistive factor. Our magnetic actuation system is engineered to generate sufficient gradient forces to overcome the predicted drag in target vessels, where flow velocities typically range from approximately 1 to 5 mm/s. Beyond forward propulsion, achieving precise manipulation in such a dynamic flow demands specific stabilization strategies. One effective method involves applying a static, non-rotating magnetic field during pauses between active manipulation steps. This field exerts a stabilizing torque that aligns and holds the actuator in a fixed orientation relative to the flow. Depending on the actuator's geometry and its contact with the vessel wall or a thrombus, this magnetic anchoring can effectively counteract drift, enabling accurate positional control for subsequent tasks.

The challenge is further compounded by the tortuous geometry of the microvascular network itself. Bends, bifurcations, and irregularities in the vessel path cause significant local variations in both flow velocity and direction. At these features, asymmetric fluid forces and secondary flow patterns can act upon the actuator, potentially deflecting it from its intended trajectory. To navigate this complex environment reliably, the magnetic control system must be capable of dynamic compensation for such transient perturbations. In our ongoing work, we are developing adaptive control strategies designed to respond to these environmental variations in real time. By integrating positional feedback, potentially from imaging modalities, the system can adjust magnetic field parameters—such as gradient, direction, and frequency—to correct deviations and maintain precise navigation along a pre-planned or dynamically determined vascular path.

It is important to recognize that convective flow is not solely an obstacle; it can also be leveraged to advantage in specific operational scenarios. For instance, during mechanical interventions such as thrombus fragmentation, the natural flow can aid in clearing generated debris from the working site. This helps maintain a clear interface for visualization and continued manipulation, potentially improving procedural outcomes. This multifaceted perspective—viewing blood flow as a variable environmental factor that presents both constraints and opportunities—is central to advancing the in vivo application of our platform. A thorough consideration of these hemodynamic factors, from fundamental fluid-structure interactions to the requirements for adaptive control, is therefore essential for translating articulated magnetic actuators from controlled laboratory settings to functional operation within the living circulatory system.

Supporting Figures

**Figure S1.** Magnetic field frequency-dependent locomotion of paramagnetic cylinders (Ø0.5 × 1 mm) under fixed rotating field amplitude (Bm = 5 mT).

**Figure S2.** Slope-climbing performance of paramagnetic cylinders (Ø0.5 × 1 mm) under 5 mT, 5 Hz rotating magnetic field.

**Figure S3.** Magnetic flux density-dependent locomotion efficiency of paramagnetic cylinders (Ø0.5 × 1 mm) at fixed excitation frequency (1 Hz)


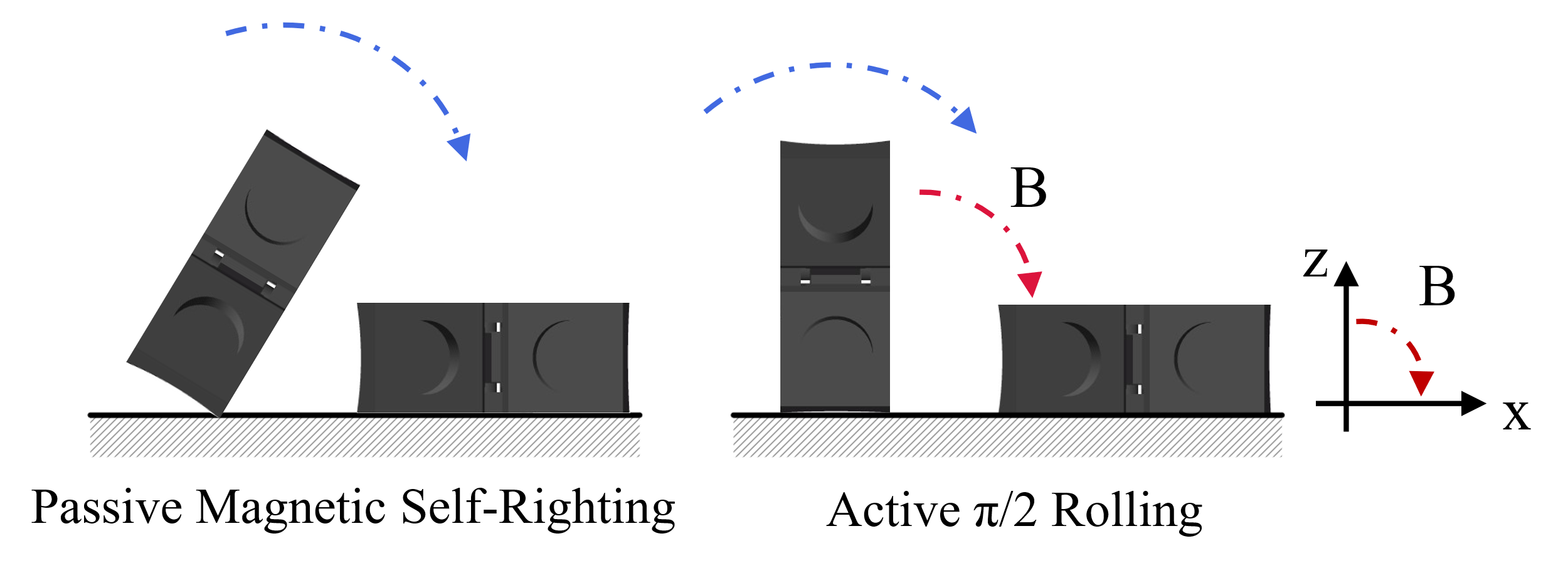


**Figure S4.** Passive realignment and active posture resetting.


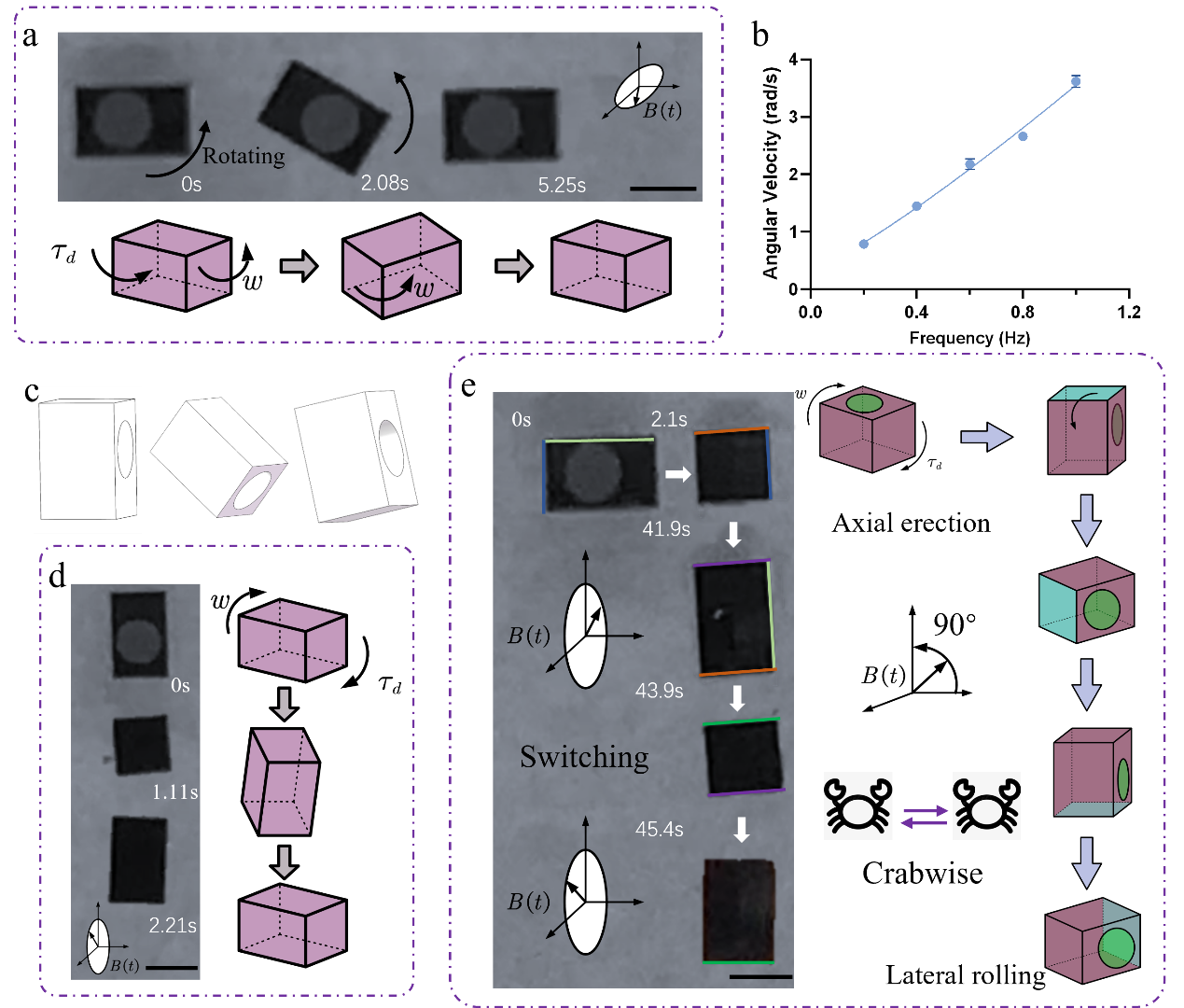


**Figure S5.** Motion characteristics and mode transitions of the modular component. (a) In-plane rotational motion of the module under rotating magnetic fields. (b) Rotation frequency corresponds directly to the applied field frequency. (c) Standard components with embedded magnetic disks in varying positions (offset, bottom, center). Dimensions: 3cm (Length) × 2cm (Width) × 2cm (Height). (d) Forward tumbling locomotion along the magnetic field direction. (e) Lateral translation capability. Scale bars, 2 mm.


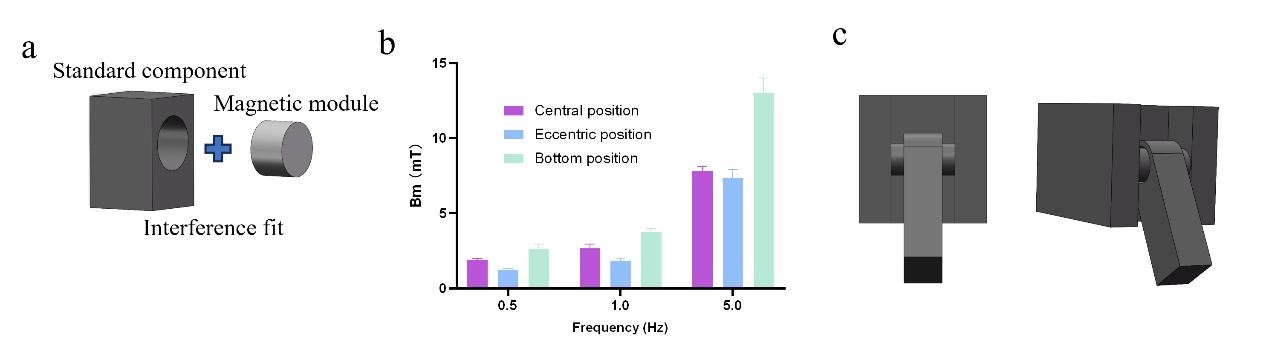


**Figure S6.** Standard systems. (a) Fundamental assembly configuration of the standard module. (b) Minimum required magnetic flux density for transportation at varying frequencies and spatial positions. (c) Revolute joint-integrated standard components, illustrating dual perspectives. Internal/external clearance of revolute joint: 0.2mm.


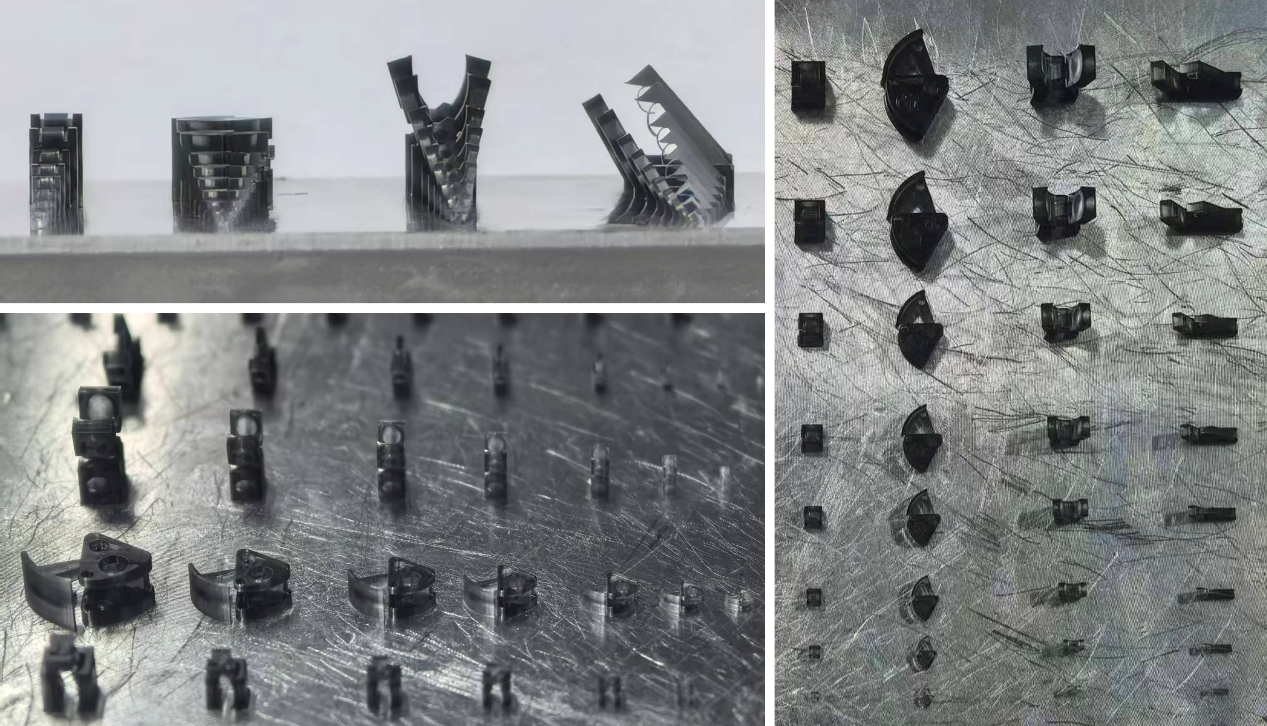


**Figure S7.** Fabricated prototypes of the four actuator designs at various sizes from different perspectives.


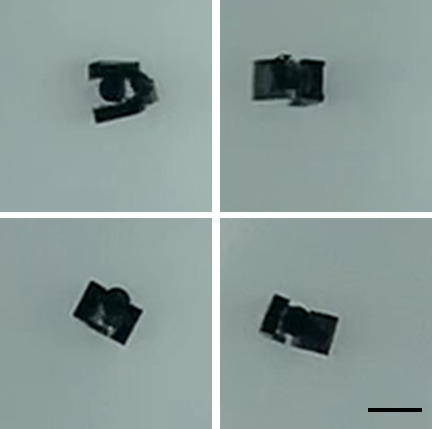


**Figure S8.** Instability in wide-angle Magnetic Tweezer (MT): Partial opening due to remanent magnetic moments upon field removal prevents stable gripping without object release, rendering MT inactive for object manipulation. Scale bars, 5 mm.


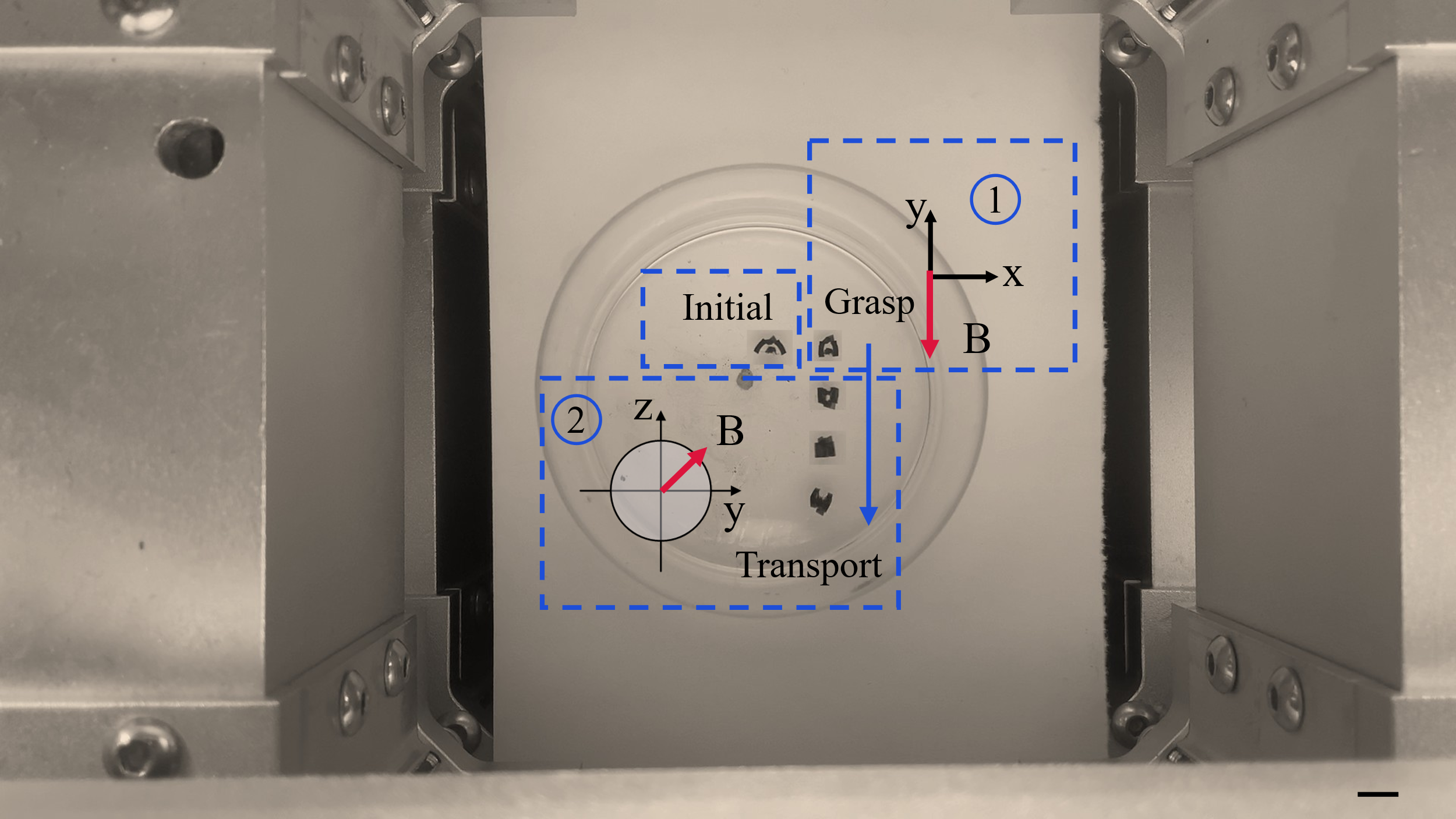


**Figure S9.** Active cargo capture and transport using the Magnetic Tweezer (MT). Illustration of the key steps of the active grasping process. (i) The MT is initially positioned near a target cargo particle with its arms in the open state. (ii) Upon application of a static magnetic field, the tweezer arms close to securely grasp the cargo. (iii) Switching the actuation to a rotating magnetic field initiates rolling locomotion of the MT while the cargo remains firmly enclosed, demonstrating combined capture and transport functionality. The experiment was conducted via manual operation based on visual feedback. Scale bars, 5 mm.


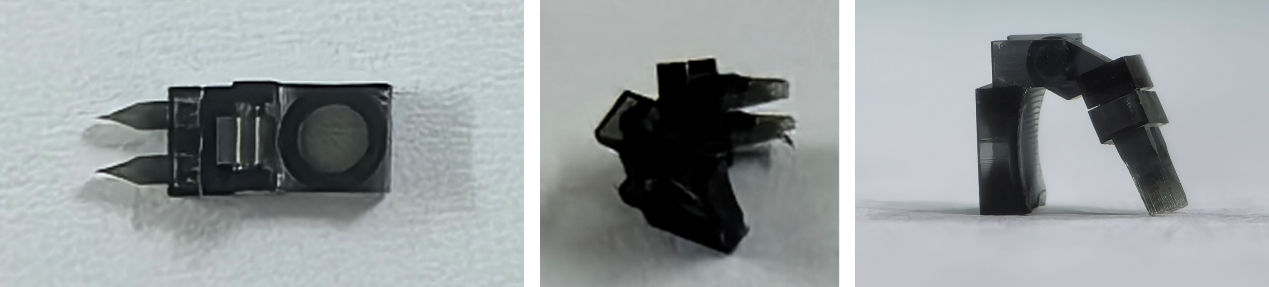


**Figure S10.** Multiple forms of the modified MM: fully extended; curled up; standing.


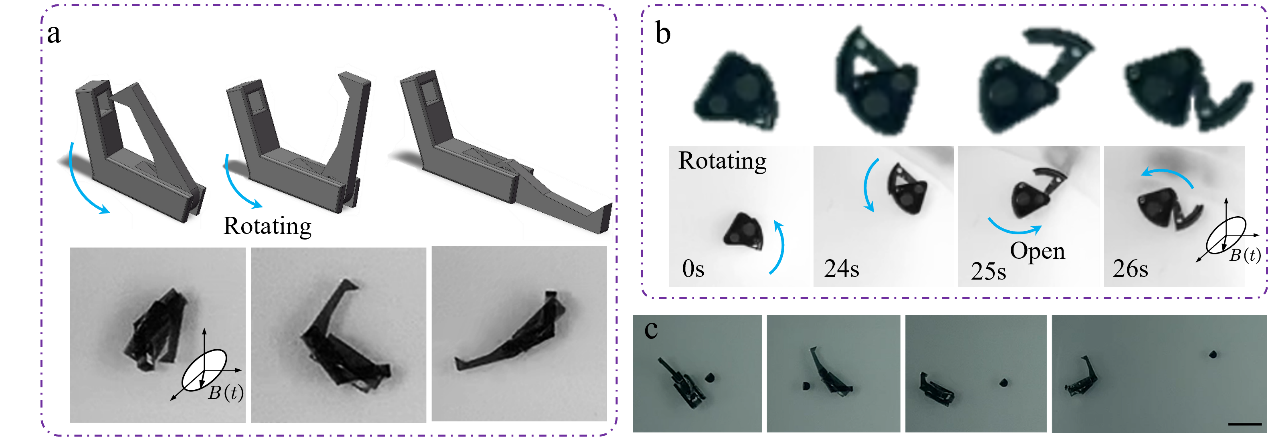


**Figure S11.** Functional switching and locomotion of the MM and MP actuators. (a) In-plane rotational motion of MM for functional manipulation. (b) MP aperture control through field-driven rotation. (c) MM exiting the workspace after object release. Exit in release mode, top view. Scale bars, 5 mm.


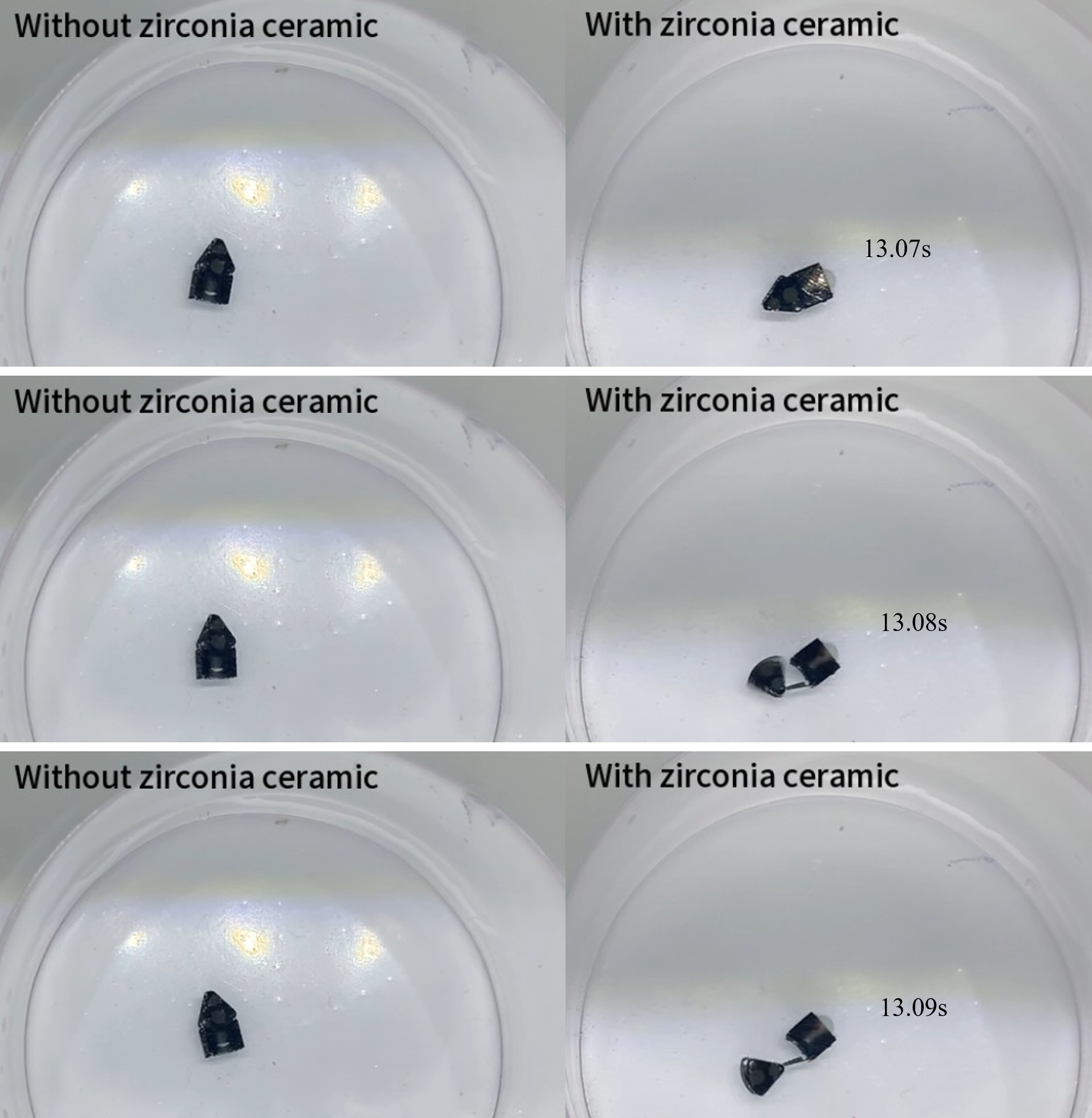


**Figure S12.** Lid‑opening dynamics of lightweight and high‑inertia MP variants. Side‑by‑side snapshots compare the magnetic‑triggered opening behavior of the standard lightweight design (left) and the zirconia‑weighted high‑inertia variant (right). The lightweight lid exhibits delayed and incomplete opening under the same rotational field, whereas the high‑inertia variant achieves full opening within 0.02 s after field application


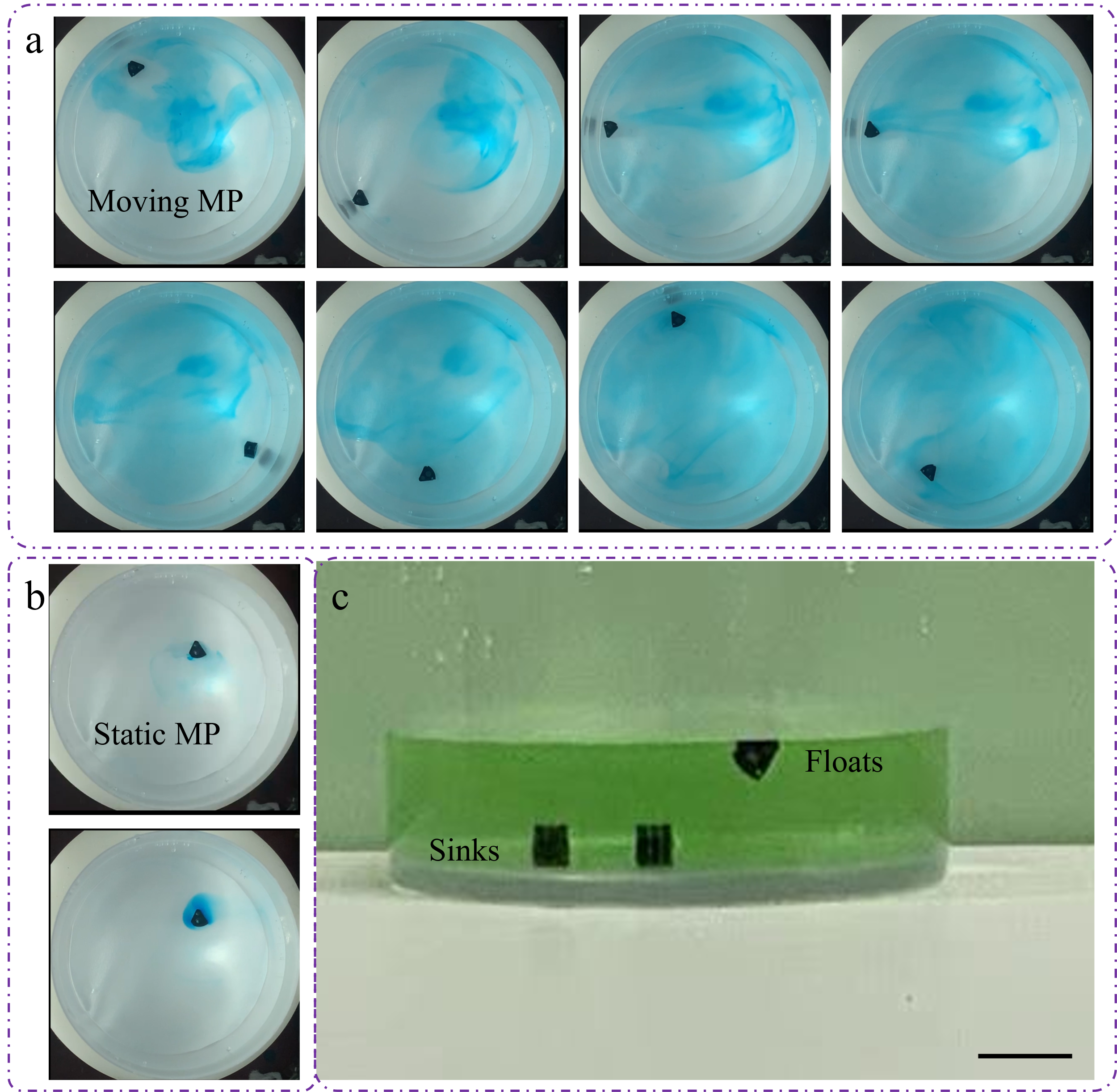


**Figure S13.** Functional characterization of the MP. (a) Time-lapse sequence (30 s intervals, left to right) of motion-assisted liquid dispersion. The MP’s active locomotion enhances payload diffusion. Scale bar, 20 mm. (b) Passive liquid diffusion amplitude from a static MP under quiescent conditions. The MP remains stationary, demonstrating diffusion solely from a fixed source. Scale bar, 20 mm. (c) Sealing performance comparison. The well-sealed, baffled MP (right) remains afloat at the air-water interface, while the unbaffled version (left) sinks. Scale bars, 15 mm.


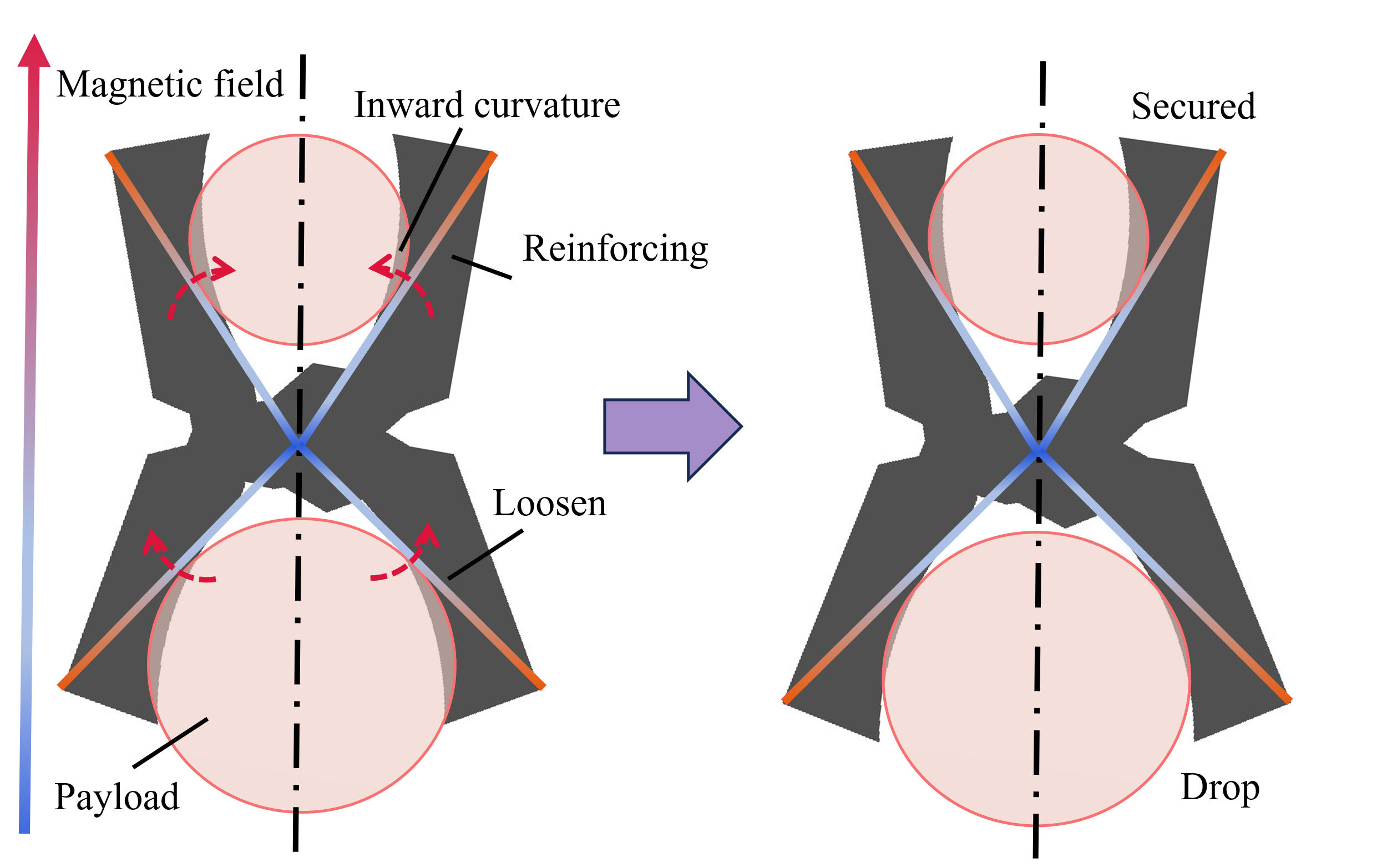


**Figure S14.** Schematic of the Stage 1of the sequential release mechanism in the Magnetic Clip. A static magnetic field creates an opening torque on the Target Arm (down) by exploiting the programmed angular offset between the magnetic moments of the two arms, reducing force to release Payload 1. The Safe Arm (up) experiences a closing torque, maintaining its grip on Payload 2.


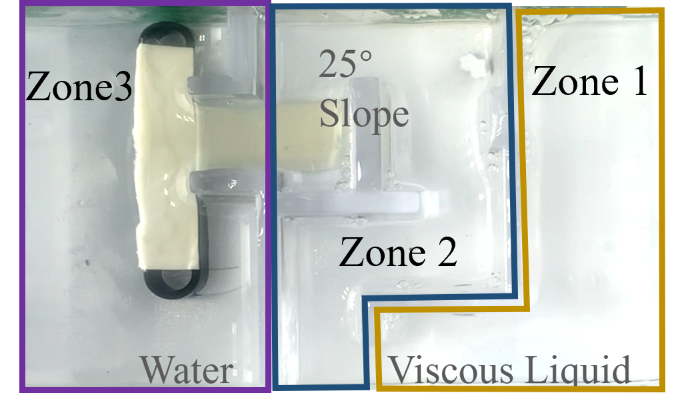


**Figure S15.** Testing platform for sequential release protocols.


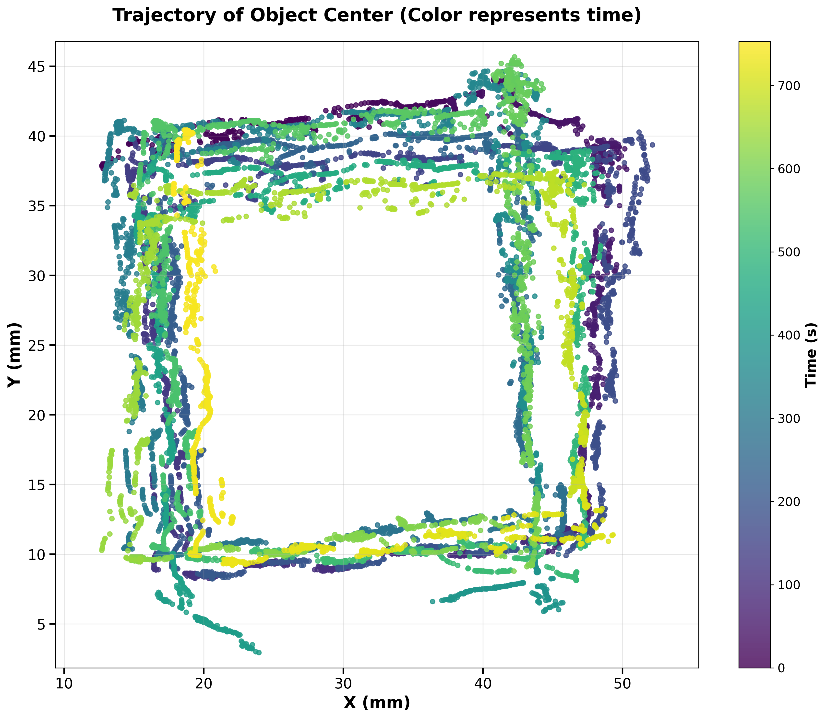


**Figure S16.** Precision of trajectory following. Path-tracking performance of the MT (body length = 7 mm) evaluated under controlled stationary fluid conditions. The robot was commanded to follow a square reference trajectory over seven consecutive cycles. The plot shows the commanded path (dashed line) overlaid with the actual recorded trajectories (solid lines), demonstrating high repeatability. The root-mean-square error (RMSE) across all cycles is approximately 2.0 mm, corresponding to less than 0.3 body lengths, confirming precise and drift-robust pose control during locomotion.


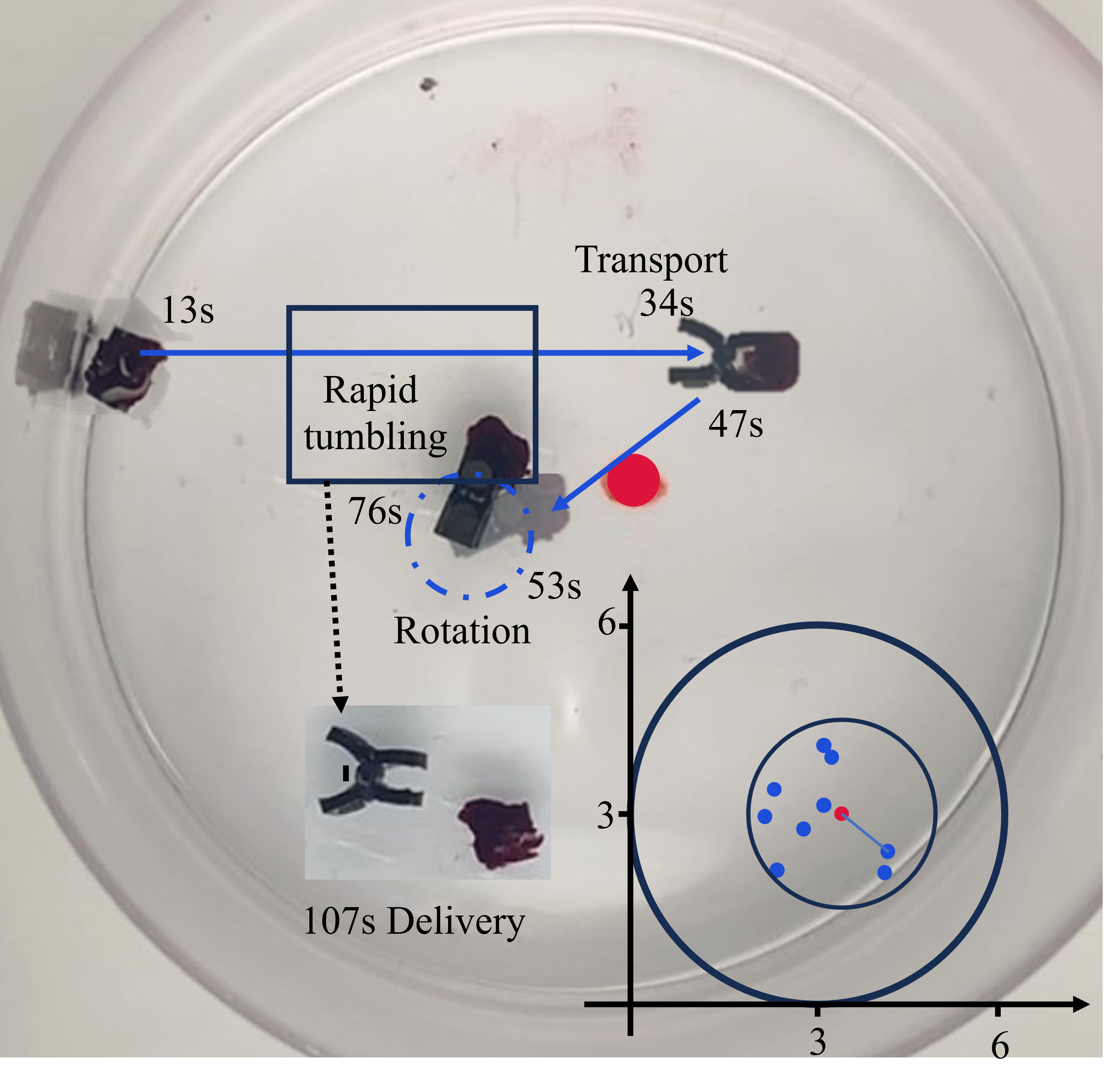


**Figure S17.** Accuracy of the dynamic tumbling release mechanism. Spatial accuracy of targeted payload delivery quantified over nine independent trials in a controlled fluid environment. The release was executed by navigating the MC to the target vicinity and performing a rapid back-and-forth tumbling maneuver to dislodge the cargo. The scatter plot shows the final positions of the released payload relative to the target center (marked as a cross). The average displacement from the target is approximately 1.0 cm (about 1.4 body lengths), demonstrating reliable sub-centimeter placement accuracy suitable for meso-scale biomedical applications.


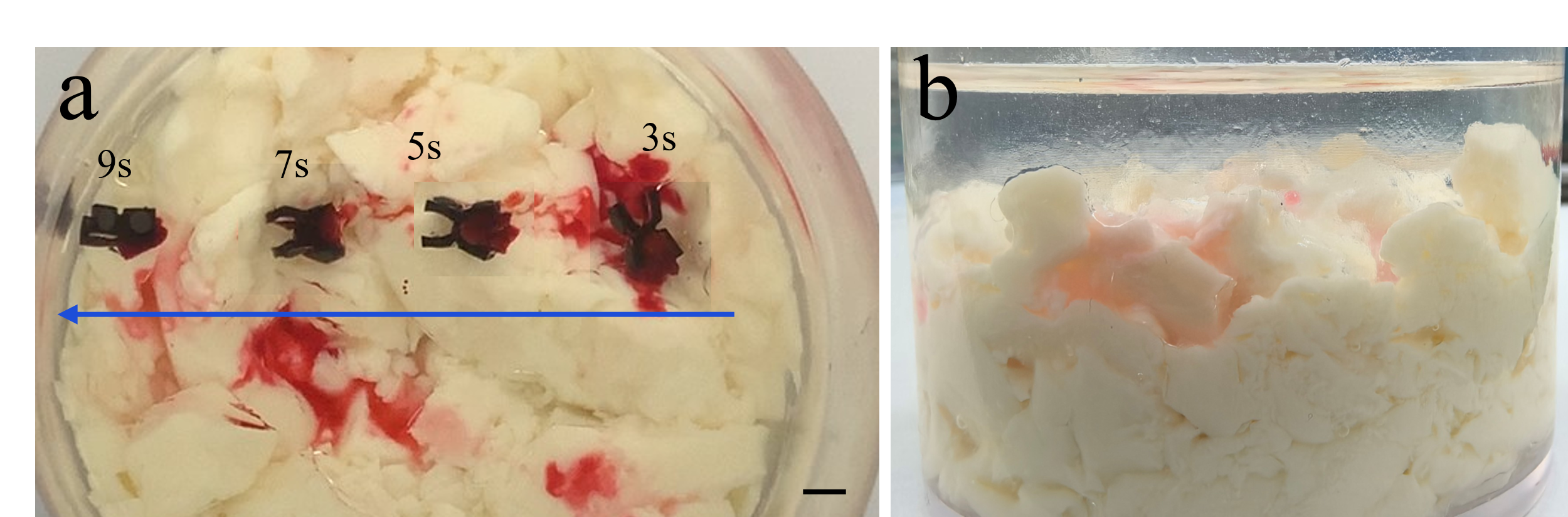


**Figure S18.** Traversal of complex, biomimetic soft terrain by the MC. Scale bars, 3 mm.

**Supporting Movies**

Movie S1. MT stable grasping with instantaneous spring-actuated detachment upon field removal;

Movie S2. MT stable grasping, magnetically actuated high-speed rotation and centrifugal discharge;

Movie S3. Active cargo capture and transport using the Magnetic Tweezer (MT).

Movie S4. Micro MT locomotion through tube.

Movie S5. MM transporting and releasing with mode switching;

Movie S6. MP mode switching;

Movie S7. MP liquid transport with position-specific rotational diffusion of dye;

Movie S8. Lid‑opening dynamics of lightweight and high‑inertia MP variants.

Movie S9. MC achieves programmed release.

Movie S10. MC achieves sequence release.

Movie S11. the MC's intelligent delivery proficiency in complex environments via a multistage magnetic field modulation strategy;

Movie S12. Example of targeted cargo delivery by the MC.

Movie S13. MC performing targeted cargo delivery on the surface of an *ex vivo* porcine stomach.

Movie S14. Traversal of complex, biomimetic soft terrain by the MC.
